# Tau PET positivity in individuals with and without cognitive impairment varies with age, amyloid-β status, *APOE* genotype and sex

**DOI:** 10.1038/s41593-025-02000-6

**Published:** 2025-07-16

**Authors:** Rik Ossenkoppele, Emma M. Coomans, Liana G. Apostolova, Suzanne L. Baker, Henryk Barthel, Thomas G. Beach, Tammy L. S. Benzinger, Tobey Betthauser, Gérard N. Bischof, Michel Bottlaender, Pierick Bourgeat, Anouk den Braber, Matthias Brendel, Adam M. Brickman, David M. Cash, Maria C. Carrillo, William Coath, Bradley T. Christian, Brad C. Dickerson, Vincent Dore, Alexander Drzezga, Azadeh Feizpour, Wiesje M. van der Flier, Nicolai Franzmeier, Giovanni B. Frisoni, Valentina Garibotto, Elsmarieke van de Giessen, Juan Domingo-Gispert, Johannes Gnoerich, Yuna Gu, Yihui Guan, Bernard J. Hanseeuw, Theresa M. Harrison, Clifford R. Jack, Elena Jaeger, William J. Jagust, Willemijn J. Jansen, Renaud La Joie, Keith A. Johnson, Sterling C. Johnson, Ian A. Kennedy, Jun Pyo Kim, Koen van Laere, Julien Lagarde, Patrick Lao, José A. Luchsinger, Silke Kern, William C. Kreisl, Vincent Malotaux, Maura Malpetti, Jennifer J. Manly, Xiaoxie Mao, Niklas Mattsson-Carlgren, Mayo Clinic Study on Aging, Clifford R. Jack, Ron C. Petersen, Konstantin Messerschmidt, Carolina Minguillon, Elizabeth M. Mormino, John T. O’Brien, Sebastian Palmqvist, Debora E. Peretti, Ron C. Petersen, Yolande A. L. Pijnenburg, Michael J. Pontecorvo, Judes Poirier, PREVENT-AD Research Group, Judes Poirier, Sylvia Villeneuve, Gil D. Rabinovici, Nesrine Rahmouni, Shannon L. Risacher, Pedro Rosa-Neto, Howard Rosen, Christopher C. Rowe, James B. Rowe, Michael Rullmann, Yasmine Salman, Marie Sarazin, Andrew J. Saykin, Julie A. Schneider, Michael Schöll, Jonathan M. Schott, Sang Won Seo, Geidy E. Serrano, Sergey Shcherbinin, Mahnaz Shekari, Ingmar Skoog, Ruben Smith, Reisa A. Sperling, Laure Spruyt, Erik Stomrud, Olof Strandberg, Joseph Therriault, Fang Xie, Rik Vandenberghe, Victor L. Villemagne, Sylvia Villeneuve, Pieter Jelle Visser, Hillary Vossler, Christina B. Young, Colin Groot, Oskar Hansson

**Affiliations:** 1 Clinical Memory Research Unit, Department of Clinical Sciences in Malmö, Lund University, Lund, Sweden; 2 Alzheimer Center Amsterdam, Neurology, Amsterdam UMC; location VUmc, Amsterdam, The Netherlands; 3 Neurodegeneration, Amsterdam Neuroscience, Amsterdam, The Netherlands; 4 Indiana University School of Medicine, Indianapolis, IN USA; 5 Lawrence Berkeley National Laboratory, Berkeley, CA USA; 6 Department of Nuclear Medicine, University Hospital Leipzig, Leipzig, Germany; 7 Banner Sun Health Research Institute, Sun City, AZ USA; 8 Department of Radiology, Washington University School of Medicine, St. Louis, MO USA; 9 Charles F. and Joanne Knight Alzheimer Disease Research Center, Washington, Washington University School of Medicine, St. Louis, MO USA; 10 Wisconsin Alzheimer’s Institute, School of Medicine and Public Health, University of Wisconsin-Madison, Madison, WI USA; 11 Wisconsin Alzheimer’s Disease Research Center, School of Medicine and Public Health, University of Wisconsin-Madison, Madison, WI USA; 12 Department of Medical Physics, University of Wisconsin-Madison, Madison, WI USA; 13 Faculty of Medicine, University of Cologne and Department of Nuclear Medicine University Hospital Cologne, Cologne, Germany; 14 Molecular Organization of the Brain, Institute for Neurosciences and Medicine, Jülich, Germany; 15 Université Paris-Saclay, BioMaps, Service Hospitalier Frédéric Joliot CEA, CNRS, INSERM, Orsay, France; 16 UNIACT, Neurospin, CEA, Gif-sur-Yvette, France; 17 Australian eHealth Research Centre, CSIRO, Melbourne, Victoria Australia; 18 Department of Biological Psychology, Vrije Universiteit Amsterdam, Amsterdam, The Netherlands; 19 Department of Nuclear Medicine, LMU Hospital, Ludwig-Maximilians-University of Munich, Munich, Germany; 20 German Center for Neurodegenerative Diseases (DZNE), Munich, Germany; 21 Gertrude H. Sergievsky Center and Taub Institute for Research on Alzheimer’s Disease and the Aging Brain, Vagelos College of Physicians and Surgeons, Columbia University, New York, NY USA; 22 Department of Neurology, Vagelos College of Physicians and Surgeons, Columbia University, New York, NY USA; 23 Dementia Research Centre, UCL Queen Square Institute of Neurology, University College London, London, UK; 24 UK Dementia Research Institute at University College London, London, UK; 25 Medical & Scientific Relations, Alzheimer’s Association, Chicago, IL USA; 26 Dementia Research Centre, UCL Queen Square Institute of Neurology, London, UK; 27 Waisman Center, University of Wisconsin-Madison, Madison, WI USA; 28 Frontotemporal Disorders Unit, Department of Neurology, Massachusetts General Hospital, Harvard Medical School, Boston, MA USA; 29 Thinoula A. Martinos Center for Biomedical Imaging, Boston, MA USA; 30 Massachusetts Alzheimer’s Disease Research Center, Boston, MA USA; 31 Department of Molecular Imaging & Therapy, Austin Health, Heidelberg, Victoria Australia; 32 The Florey Institute of Neuroscience and Mental Health, The University of Melbourne, Parkville, Victoria Australia; 33 Department of Epidemiology and Biostatistics, Amsterdam UMC; location VUmc, Amsterdam, The Netherlands; 34 Munich Cluster for Systems Neurology (SyNergy), Munich, Germany; 35 Institute for Stroke and Dementia Research (ISD), University Hospital, LMU Munich, Munich, Germany; 36 Department of Psychiatry and Neurochemistry, University of Gothenburg, The Sahlgrenska Academy, Institute of Neuroscience and Physiology, Gothenburg, Sweden; 37 Memory Clinic, Geneva University Hospitals, Geneva, Switzerland; 38 Division of Nuclear Medicine and Molecular Imaging, Geneva University Hospitals, Geneva, Switzerland; 39 Laboratory of Neuroimaging and Innovative Molecular Tracers (NIMTlab), Geneva University Neurocenter and Faculty of Medicine, University of Geneva, Geneva, Switzerland; 40 CIBM Center for Biomedical Imaging, Geneva, Switzerland; 41 Department of Radiology & Nuclear Medicine, Amsterdam UMC; location VUmc, Amsterdam, The Netherlands; 42 Barcelonaβeta Brain Research Center (BBRC), Pasqual Maragall Foundation, Barcelona, Spain; 43 Centro de Investigación Biomédica en Red de Bioingeniería, Biomateriales y Nanomedicina, Madrid, Spain; 44 Department of Neurology, Samsung Medical Center, Sungkyunkwan University School of Medicine, Seoul, Republic of Korea; 45 Department of Nuclear Medicine & PET Center, Huashan Hospital, Fudan University, Shanghai, China; 46 Institute of Neuroscience, UCLouvain, Brussels, Belgium; 47 Department of Neurology, Cliniques Universitaires Saint-Luc, Brussels, Belgium; 48 Gordon Center for Medical Imaging, Department of Radiology, Mass General Brigham, Boston, MA USA; 49 Department of Neuroscience, University of California Berkeley, Berkeley, CA USA; 50 Department of Radiology, Mayo Clinic, Rochester, MN USA; 51 Department of Nuclear Medicine, University of Cologne, University Hospital of Cologne, Cologne, Germany; 52 Alzheimer Center Limburg, School for Mental Health and Neuroscience, Maastricht University, Maastricht, The Netherlands; 53 Memory and Aging Center, Department of Neurology, University of California, San Francisco, CA USA; 54 Department of Neurology, Massachusetts General Hospital, Harvard Medical School, Boston, MA USA; 55 Center for Alzheimer Research and Treatment, Department of Neurology, Brigham and Women’s Hospital, Harvard Medical School, Boston, MA USA; 56 Athinoula A. Martinos Center for Biomedical Imaging, Department of Radiology, Massachusetts General Hospital, Harvard Medical School, Boston, MA USA; 57 Department of Radiology, Massachusetts General Hospital, Harvard Medical School, Boston, MA USA; 58 Eli Lilly and Company, Indianapolis, IN USA; 59 Department of Imaging and Pathology, Nuclear Medicine and Molecular Imaging, KU Leuven, Leuven, Belgium; 60 Division of Nuclear Medicine, University Hospitals Leuven, Leuven, Belgium; 61 Department of Neurology of Memory and Language, GHU Paris Psychiatrie & Neurosciences, Hôpital Sainte Anne, Paris, France; 62 Department of Neurology, College of Physicians and Surgeons, Columbia University, New York, NY USA; 63 Departments of Medicine and Epidemiology, Columbia University Irving Medical Center, New York, NY USA; 64 Department of Psychiatry and Neurochemistry, Institute of Neuroscience and Physiology, Sahlgrenska Academy, University of Gothenburg, Mölndal, Sweden; 65 Department of Neuropsychiatry, Sahlgrenska University Hospital, Gothenburg, Sweden; 66 Department of Clinical Neurosciences, University of Cambridge, Cambridge, UK; 67 Gertrude H. Sergievsky Center and Taub Institute for Research on Alzheimer’s Disease and the Aging Brain, College of Physicians and Surgeons, Columbia University, New York, NY USA; 68 Department of Neurology, Skåne University Hospital, Lund University, Lund, Sweden; 69 Wallenberg Center for Molecular Medicine, Lund University, Lund, Sweden; 70 Department of Neurology and Neurological Sciences, Stanford University School of Medicine, Stanford, CA USA; 71 Department of Psychiatry, University of Cambridge School of Clinical Medicine, Cambridge, UK; 72 Memory Clinic, Skåne University Hospital, Malmö, Sweden; 73 Department of Neurology, Mayo Clinic, Rochester, MN USA; 74 Douglas Mental Health University Institute, Centre for Studies on the Prevention of Alzheimer’s Disease (StoP-AD), Montréal, Québec Canada; 75 Department of Psychiatry, McGill University, Montréal, Québec Canada; 76 Department of Radiology & Biomedical Imaging, University of California, San Francisco, CA USA; 77 Translational Neuroimaging Laboratory, McGill Research Centre for Studies in Aging, Montréal, Québec Canada; 78 Department of Neurology and Neurosurgery, Faculty of Medicine, McGill University, Montréal, Québec Canada; 79 Indiana University, Indianapolis, IN USA; 80 Cambridge University Hospitals NHS Foundation Trust, Cambridge, UK; 81 Medical Research Council Cognition and Brain Sciences Unit, University of Cambridge, Cambridge, UK; 82 Clinic for Cognitive Neurology, University Hospital of Leipzig and Max Planck Institute for Human Cognitive and Brain Sciences, Leipzig, Germany; 83 Department of Neurology of Memory and Language, GHU Paris Psychiatrie & Neurosciences, Hôpital Sainte-Anne, Paris, France; 84 Rush Alzheimer’s Disease Center, Rush University Medical Center, Chicago, IL USA; 85 Department of Neurological Sciences, Rush University Medical Center, Chicago, IL USA; 86 Department of Pathology, Rush University Medical Center, Chicago, IL USA; 87 Wallenberg Centre for Molecular and Translational Medicine, University of Gothenburg, Gothenburg, Sweden; 88 Department of Psychiatry and Neurochemistry, University of Gothenburg, Mölndal, Sweden; 89 Dementia Research Centre, Queen Square Institute of Neurology, University College London, London, UK; 90 UK Dementia Research Institute, London, UK; 91 Laboratory for Cognitive Neurology, Department of Neurosciences, Leuven Brain Institute, KU Leuven, Leuven, Belgium; 92 Neurology Service, University Hospital Leuven, Leuven, Belgium; 93 Department of Psychiatry, University of Pittsburgh, Pittsburgh, PA USA

**Keywords:** Alzheimer's disease, Diagnostic markers

## Abstract

Tau positron emission tomography (PET) imaging allows in vivo detection of tau proteinopathy in Alzheimer’s disease, which is associated with neurodegeneration and cognitive decline. Understanding how demographic, clinical and genetic factors relate to tau PET positivity will facilitate its use for clinical practice and research. Here we conducted an analysis of 42 cohorts worldwide (*N* = 12,048), including 7,394 cognitively unimpaired (CU) participants, 2,177 participants with mild cognitive impairment (MCI) and 2,477 participants with dementia. We found that from age 60 years to 80 years, tau PET positivity in a temporal composite region increased from 1.1% to 4.4% among CU amyloid-β (Aβ)-negative participants and from 17.4% to 22.2% among CU Aβ-positive participants. Across the same age span, tau PET positivity decreased from 68.0% to 52.9% in participants with MCI and from 91.5% to 74.6% in participants with dementia. Age, Aβ status, *APOE* ε4 carriership and female sex were all associated with a higher prevalence of tau PET positivity across groups. *APOE* ε4 carriership in CU individuals lowered the age at onset of both Aβ positivity and tau positivity by decades. Finally, we replicated these associations in an independent autopsy dataset (*N* = 5,072 from 3 cohorts).

## Abstract (web summary)

Ossenkoppele, Coomans and colleagues analyzed the tau PET data of 12,048 individuals from 42 cohorts worldwide. They found that age, amyloid-β status, presence of an *APOE* ε4 allele and female sex are key contributors to tau PET positivity, which should aid clinical decision-making and trial designs.

## Main

Alzheimer’s disease (AD) is the most common cause of dementia, with a worldwide prevalence of ~32 million in 2023, which is expected to double by 2060 because of increased life expectancy^1^. AD is neuropathologically characterized by the aggregation of amyloid-β (Aβ) proteins into extracellular plaques and of tau proteins into intracellular neurofibrillary tangles. Since the 2000s (Aβ)^2^ and 2010s (tau)^3^, both proteinopathies can be visualized and quantified in the living human brain using positron emission tomography (PET). This has led to pivotal insights into the progression of AD over time. For example, amyloid-PET studies have consistently shown that Aβ proteinopathy is an early event in the AD pathophysiological process and typically emerges decades before symptom onset^4^. As such, many elderly cognitively unimpaired (CU) individuals exhibit considerable Aβ proteinopathy without manifest cognitive deficits (that is, at age 70 years, PET-assessed Aβ positivity is ~23%, which increases to ~48% when carrying at least one *APOE* ε4 allele)^5,6^. Consequently, the temporal association between Aβ proteinopathy and cognitive decline is moderate^7,8^. Also, major reductions of Aβ proteinopathy achieved by monoclonal antibody therapy have led to statistically significant but modest clinical benefits in symptomatic AD^9,10^. In contrast, the presence and amount of tau proteinopathy are strongly associated with neurodegeneration, cognitive impairment, rate of clinical progression and treatment response to amyloid-lowering therapies^11,12^. Even in CU individuals, the presence of tau proteinopathy as measured by PET profoundly increases the risk of short-term clinical progression^13,14^.

Based on the recognition of tau proteinopathy as a key manifestation of AD, tau PET tracers are increasingly used in both the clinic and trials^15^ and are now incorporated into the core diagnostic criteria for AD^16,17^. One tau PET tracer (that is, [^18^F]flortaucipir/Tauvid) has received approval from the US Food and Drug Administration for clinical use to support a clinical diagnosis of AD^18^, because it (and other tau PET tracers) can accurately distinguish between AD dementia and most other (non-AD) neurodegenerative disorders^19,20^. Furthermore, several tau PET tracers have been implemented into clinical trials for participant selection, stratification and/or as a secondary or exploratory outcome measure. This includes application in anti-tau trials^21,22^, but also in anti-Aβ trials^9,10^. To optimize the future use of tau PET in clinical settings, accurate prevalence estimates of tau PET positivity and understanding of how demographic, clinical and genetic factors are associated with these prevalence estimates are essential. This will help clinicians and trialists to interpret the clinical importance of tau PET results and inform clinical trial design. Most tau PET studies conducted to date are single-center studies with insufficient sample sizes for providing reliable prevalence estimates of tau PET positivity, especially when these samples are stratified to explore the effects of individual risk factors for AD-type dementia such as age, sex and *APOE* genotype.

In the present study, we conducted a large-scale, multicenter analysis of 42 cohorts worldwide (*N* = 12,048). The present study aimed to estimate the prevalence of tau proteinopathy as measured by PET in CU participants and in individuals with mild cognitive impairment (MCI) or dementia. We investigated whether and how Aβ positivity, age, sex and *APOE* genotype are associated with tau PET-positivity prevalence estimates. We also compared the estimated tau PET-positivity prevalence against gold standard assessment of tau pathology, that is, the prevalence of neocortical tau proteinopathy in an independent postmortem dataset (*n* = 5,072).

## Results

We included 12,048 participants with tau PET from 42 cohorts worldwide, of whom 7,394 were CU participants (mean age: 68.7 ± 11.1 years, 55.9% women, 30.9% Aβ positive), 2,177 with MCI (mean age: 71.3 ± 8.8, 45.0% women, 59.3% Aβ positive) and 2,477 with dementia (mean age: 69.9 ± 9.0, 50.9% women, 76.8% Aβ positive; Table 1). Participant characteristics stratified by Aβ status are presented in Supplementary Table 1. In addition, we included 5,072 participants from 3 independent autopsy cohorts (1,026 CU, 661 MCI and 3,385 dementia; Extended Data Table 1). Throughout the text, the term ‘tau positivity’ refers to a positive (abnormal) tau PET scan based on suprathreshold (cohort-specific threshold of mean + 2 s.d. in Aβ-negative CU individuals who were aged ≥50 years) tracer uptake in a previously established AD-specific region of interest (ROI), covering medial and lateral parts of the temporal cortex^4,19^, or the presence of Braak stage V–VI for neurofibrillary tangle pathology on neuropathological examination (that is, ‘B3’ according to the AD neuropathological scoring system^23^). In secondary analyses, we assessed tau PET positivity in alternative ROIs (entorhinal cortex and a whole-brain ROI), using alternative thresholds (mean + 1 s.d. and mean + 1.5 s.d.) and alternative methods of threshold definition (Gaussian mixture modeling; see Methods for further details). The term ‘prevalence’ refers to the frequency of tau PET positivity in the current dataset.

**Table 1:** Participant characteristics

	CU	MCI	Dementia^a^
***n***	7,394	2,177	2,477
**Age, years**	68.7 ± 11.1	71.3 ± 8.8	69.9 ± 9.0
**Sex,** ***n*** **women (%)**	4,136 (55.9)	980 (45.0)	1,255 (50.9)
***APOE*** **ε4 status,** ***n*** **carrier (%)**	2,322 (35.9)	879 (47.5)	1,096 (57.0)
**Aβ status,** ***n*** **positive (%)**	2,218 (30.9)	1,258 (59.3)	1,730 (76.8)
**Aβ modality,** ***n*** **PET (%)**	6,584 (95.3)	1,892 (90.3)	1,651 (73.3)
**Education, years**	14.7 ± 3.7	13.5 ± 4.3	13.1 ± 4.2
**MMSE**	28.7 ± 1.7	26.7 ± 2.4	20.9 ± 6.0
**Race/Ethnicity,** ***n*** **self-report (% of total)**			
Non-Hispanic white	3,744 (80.5)	879 (80.8)	603 (72.1)
Asian	276 (5.9)	131 (12.0)	187 (22.4)
Black/African American	286 (6.2)	47 (4.3)	26 (3.1)
Hispanic	317 (6.8)	27 (2.5)	14 (1.7)
American Indian or Alaskan Native	9 (0.2)	0 (0.0)	2 (0.2)
Hawaiian/Pacific Islander	1 (0.0)	0 (0.0)	0 (0.0)
More than one	13 (0.3)	3 (0.3)	3 (0.4)
Other	4 (0.1)	1 (0.1)	1 (0.1)
**Tau PET tracer,** ***n*** **(%)**			
[^18^F]flortaucipir	4,118 (55.7)	1,125 (51.7)	1,237 (49.9)
[^18^F]MK6240	2,066 (27.9)	587 (27.0)	503 (20.3)
[^18^F]RO948	1,111 (15.0)	434 (19.9)	439 (17.7)
[^18^F]PI2620	99 (1.3)	31 (1.4)	298 (12.0)

Shown are mean ± s.d. unless specified otherwise. Sex was missing for 10 participants (0.1%), *APOE* ε4 status for 1,796 participants (14.9%), Aβ status for 489 participants (4.1%), Aβ modality for 789 participants (6.5%), education for 1,114 participants (9.3%), Mini-Mental State Examination (MMSE) for 792 participants (6.6%) and race for 5,593 participants (46.4%). Aβ modality refers to the method used to determine Aβ status, which could include either PET or cerebrospinal fluid markers.

^a^Patients with a syndromic dementia diagnosis met diagnostic criteria for AD-type dementia (*n* = 1,804) or non-AD neurodegenerative disorders including FTD (*n* = 162), PSP (*n* = 141), CBS (*n* = 101), DLB (*n* = 76), PDD (*n* = 39), VaD (*n* = 32) or dementia–not otherwise specified (*n* = 122).

### Tau positivity according to diagnosis and Aβ status

**Extended Data Fig. 1:**
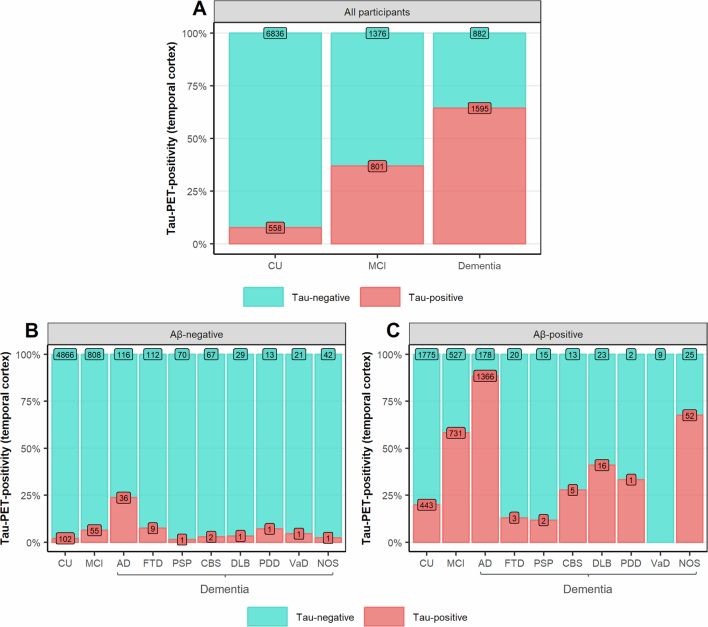
Observed tau-positivity in the temporal cortex across diagnostic groups. Plots show the observed rates of Tau-PET positivity in the temporal cortex by (syndromic and clinical) diagnosis for all participants (panel **a**, *n* = 1,2048) or stratified by Aβ-status (panels **b** [*n* = 6,353] and **c** [*n* = 5,206]). Aβ = Amyloid-beta; AD = Alzheimer’s disease, CBS = Corticobasal syndrome, DLB = Dementia with Lewy bodies, FTD = Frontotemporal dementia, MCI = Mild cognitive impairment, NOS = Not otherwise specified, PDD = Parkinson’s disease dementia, PSP = Progressive supranuclear palsy, VaD = Vascular dementia.

The observed prevalence of tau PET positivity in the temporal cortex was 7.6% (558 of 7,394) in CU individuals, 36.8% (801 of 2,177) in participants with MCI and 64.4% (1,595 of 2,477) in participants with all-cause dementia (Extended Data Fig. 1a). When stratifying for Aβ status and syndrome diagnosis, the prevalence of tau positivity in the temporal cortex was 2.1% (102 of 4,968) in Aβ-negative CU participants versus 20.0% (443 of 2,218) in Aβ-positive CU participants, 6.4% (55 of 863) in Aβ-negative participants with MCI versus 58.1% (731 of 1,258) in Aβ-positive participants with MCI and 10.0% (52 of 522) in participants with Aβ-negative dementia versus 83.5% (1,445 of 1,730) in participants with Aβ-positive dementia (Extended Data Fig. 1b,c). When stratifying for Aβ status and clinical dementia diagnosis, the prevalence of tau positivity in the temporal cortex was 23.7% (36 of 152) and 88.5% (1,366 of 1,544) for Aβ-negative versus Aβ-positive participants with AD-type dementia, 7.4% (9 of 121) and 13.0% (3 of 23) for Aβ-negative versus Aβ-positive participants with frontotemporal dementia (FTD), 1.4% (1 of 71) and 11.8% (2 of 17) for Aβ-negative versus Aβ-positive participants with progressive supranuclear palsy (PSP), 2.9% (2 of 69) and 27.8% (5 of 18) for Aβ-negative versus Aβ-positive participants with corticobasal syndrome (CBS), 3.3% (1 of 30) and 41.0% (16 of 39) for Aβ-negative versus Aβ-positive participants with dementia with Lewy bodies (DLB), 7.1%% (1 of 14) and 33.3% (1 of 3) for Aβ-negative versus Aβ-positive participants with Parkinson’s disease dementia (PDD), 4.6% (1 of 22) and 0.0% (0 of 9) for Aβ-negative versus Aβ-positive participants with vascular dementia (VaD) and 2.3% (1 of 43) and 67.5% (52 of 77) for Aβ-negative versus Aβ-positive participants with dementia–not otherwise specified (Extended Data Fig. 1b,c). The observed prevalence of tau positivity in the entorhinal cortex and a whole-brain ROI by (syndrome and clinical) diagnosis and Aβ status is presented in Supplementary Figs. 1 and 2, respectively.

### Tau positivity according to age and Aβ status

**Fig. 1:**
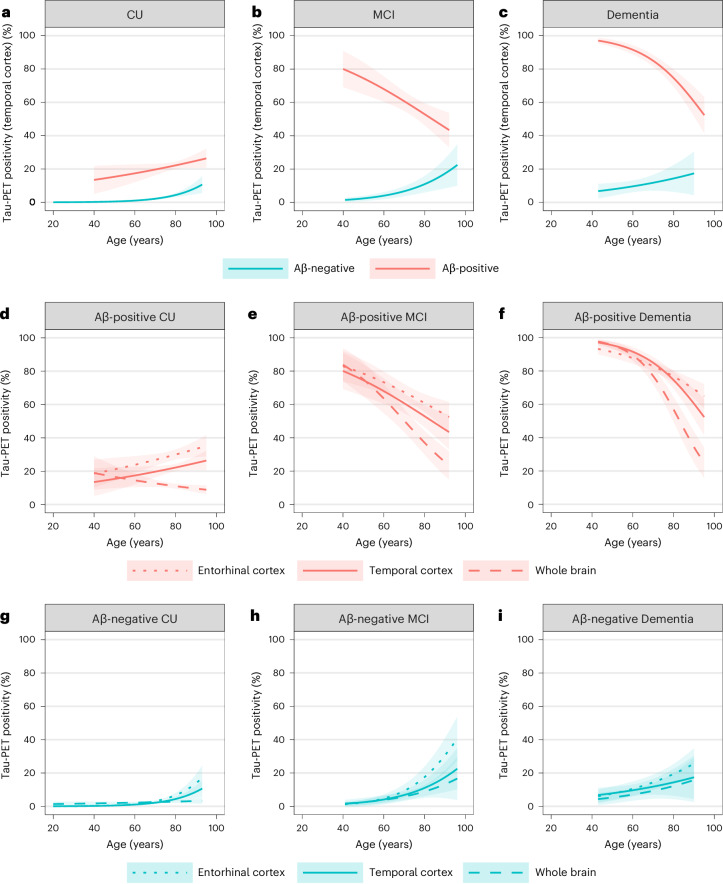
Prevalence estimates of tau PET positivity according to age, Aβ and cognitive status. **a**–**i**, Tau PET positivity in CU (**a**,**d**,**g**), MCI (**b**,**e**,**h**) and dementia (**c**,**f**,**i**) modeled using age, Aβ status and an interaction between age and Aβ status as determinants. Models were stratified by syndrome diagnosis. Tau PET positivity was assessed in the temporal cortex (**a**–**i**) as well as in the entorhinal cortex and whole brain (**d**–**i**). The figure includes 7,186 CU, 2,121 MCI and 2,252 dementia participants for tau PET positivity in the temporal cortex and whole-brain region and 7,174 CU, 2,117 MCI and 2,234 dementia participants for tau PET positivity in the entorhinal cortex. The *y* axes reflect estimated probabilities of tau PET positivity (prevalence estimates) generated from GEEs. Shaded areas indicate the 95% CIs.

**Extended Data Fig. 2:**
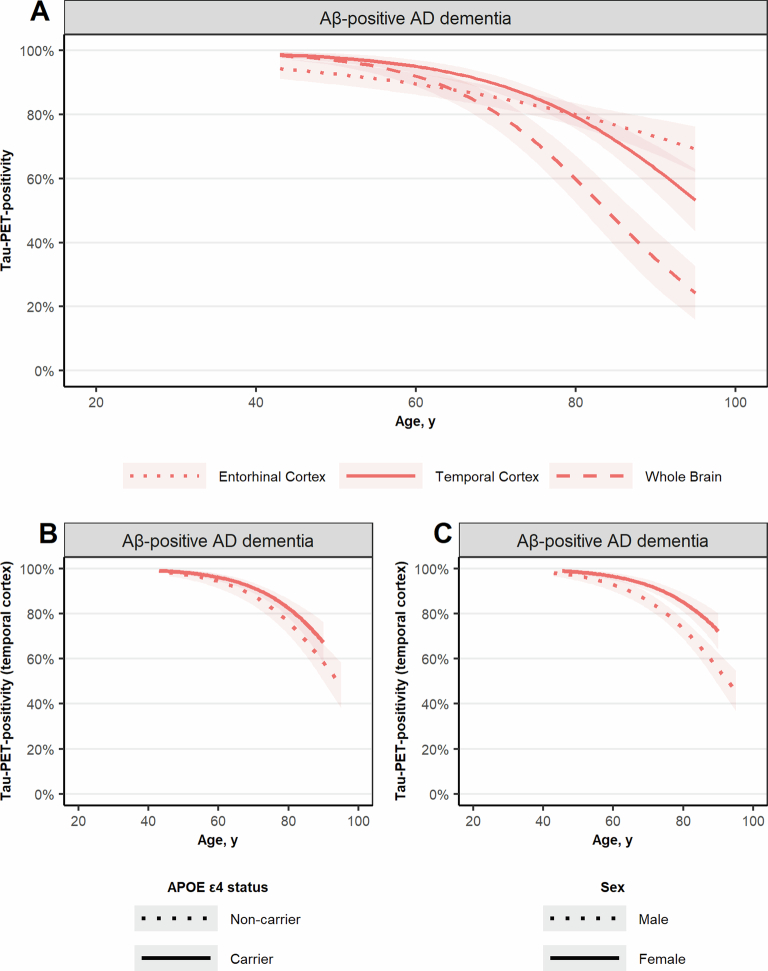
Prevalence estimates of Tau-PET positivity according to age, Aβ, cognitive status and sex in AD-type dementia. Panel **a** resembles Fig. 1f, panel **b** resembles Fig. 3c and panel **c** resembles Fig. 3f, but now in individuals clinically diagnosed with AD-type dementia instead of all-cause dementia. The model presented in panel **a** included age, Aβ-status and an interaction between age and Aβ-status (*n* = 1,696). The model presented in panel **b** included age, Aβ-status, *APOE* ε4-status and an interaction between age and Aβ-status (*n* = 1,486) The model presented in panel **c** included age, Aβ-status, sex and an interaction term between age and Aβ-status (*n* = 1,696). The y-axes reflect estimated probabilities of Tau-PET-positivity (prevalence estimates) from logistic generalized estimating equations. Shading areas indicates the 95% confidence intervals. Aβ = Amyloid-beta; APOE = Apolipoprotein E; PET = Positron emission tomography.

Logistic generalized estimating equation (GEE) models showed significant interactions between age and biomarker-defined Aβ status on tau positivity in the temporal cortex in CU participants and those with MCI and dementia (*β*, the estimated regression coefficient, = −0.06 for CU, *β* = −0.09 for MCI and *β* = −0.09 for dementia, all *P* < 0.001; Fig. 1a,c). From age 60 years to 80 years, the estimated prevalence of tau positivity in the temporal cortex increased from 1.1% (95% confidence interval (CI) 0.7–1.4%) to 4.4% (95% CI 3.2–5.6%) among Aβ-negative CU participants, and from 17.4% (95% CI 12.1–22.8%) to 22.2% (95% CI 19.9–24.5%) among Aβ-positive CU participants (Fig. 1a and Table 2). Among Aβ-negative participants with MCI and dementia, from age 60 years to 80 years, the estimated prevalence of tau positivity increased from 4.1% (95% CI 1.9–6.2%) to 11.0% (95% CI 6.0–16.0%) in MCI and from 9.7% (95% CI 6.1–13.2%) to 14.4% (95% CI 6.3–22.5%) in dementia (Fig. 1b,c and Table 2). In contrast, among Aβ-positive participants with MCI and dementia, from age 60 years to 80 years, the estimated prevalence of tau positivity decreased from 68.0% (95% CI 60.4–75.6%) to 52.9% (95% CI 46.3–59.5%) in MCI and from 91.5% (95% CI 88.8–94.3%) to 74.6% (95% CI 69.4–79.7%) in dementia (Fig. 1b,c, Table 2 and Supplementary Table 2). Next, we separately assessed tau positivity in two additional ROIs, that is, the entorhinal cortex and the whole-brain ROI. Across all groups, we observed that, at the same age, the prevalence of tau positivity was highest for the entorhinal cortex, followed by the temporal cortex and then the whole-brain ROI (Fig. 1d,i and Extended Data Tables 2 and 3). For example, in Aβ-positive CU participants aged 80 years, the estimated prevalence of tau positivity was 30.0% (95% CI 26.9–33.0%) for the entorhinal cortex, 22.2% (95% CI 19.9–24.5%) for the temporal cortex and 11.0% (95% CI 9.5–12.5%) for the whole-brain ROI. An additional analysis, including individuals with clinically diagnosed AD-type dementia, yielded very similar results compared with the analysis in the all-cause dementia group (Extended Data Fig. 2 and Extended Data Table 4). The proportion of tau positivity across ROIs for early onset (age at PET < 66 years) versus late-onset (age at PET > 65 years) AD is provided in Supplementary Fig. 3 and Supplementary Table 2.

**Table 2:** Prevalence estimates of tau PET positivity in the temporal cortex according to age, Aβ and cognitive status

Age, years	CU, % (95% CI)			MCI, % (95% CI)			Dementia, % (95% CI)		
	Total	Aβ negative	Aβ positive	Total	Aβ negative	Aβ positive	Total	Aβ negative	Aβ positive
**50**	2.4 (1.6–3.1)	0.5 (0.2–0.8)	15.3 (8.3–22.4)	32.0 (24.2–39.7)	2.4 (0.8–4.1)	74.5 (64.9–84.1)	74.5 (65.2–83.9)	7.8 (4.0–11.7)	95.4 (93.3–97.6)
**55**	3.2 (2.3–4.1)	0.7 (0.4–1.1)	16.3 (10.1–22.6)	33.7 (26.7–40.6)	3.2 (1.3–5.0)	71.3 (62.7–80.0)	73.0 (64.4–81.6)	8.7 (5.1–12.3)	93.8 (91.3–96.2)
**60**	4.2 (3.2–5.2)	1.1 (0.7–1.4)	17.4 (12.1–22.8)	35.4 (29.1–41.8)	4.1 (1.9–6.2)	68.0 (60.4–75.6)	71.4 (63.6–79.3)	9.7 (6.1–13.2)	91.5 (88.8–94.3)
**65**	5.6 (4.5–6.7)	1.5 (1.1–1.9)	18.5 (14.1–22.9)	37.2 (31.3–43.1)	5.3 (2.7–7.8)	64.4 (57.9–71.0)	69.8 (62.7–76.9)	10.7 (6.8–14.6)	88.7 (85.6–91.7)
**70**	7.4 (6.2–8.7)	2.2 (1.7–2.7)	19.7 (16.3–23.1)	39.0 (33.2–44.8)	6.8 (3.7–9.8)	60.7 (54.8–66.6)	68.1 (61.5–74.7)	11.8 (7.0–16.6)	84.9 (81.5–88.4)
**75**	9.8 (8.4–11.2)	3.1 (2.4–3.8)	20.9 (18.4–23.5)	40.9 (34.9–46.9)	8.6 (4.8–12.5)	56.9 (51.0–62.7)	66.4 (60.1–72.6)	13.1 (6.8–19.3)	80.3 (76.2–84.3)
**80**	12.8 (11.1–14.4)	4.4 (3.2–5.6)	22.2 (19.9–24.5)	42.8 (36.2–49.4)	11.0 (6.0–16.0)	52.9 (46.3–59.5)	64.6 (58.3–70.9)	14.4 (6.3–22.5)	74.6 (69.4–79.7)
**85**	16.5 (14.4–18.7)	6.2 (4.0–8.4)	23.5 (20.6–26.4)	44.7 (37.2–52.2)	13.9 (7.2–20.6)	48.9 (40.9–56.9)	62.8 (56.1–69.5)	15.8 (5.4–26.2)	67.9 (61.0–74.7)
**90**	21.1 (18.1–24.1)	8.7 (5.0–12.5)	24.9 (20.7–29.1)	46.6 (38.1–55.1)	17.4 (8.5–26.3)	45.0 (35.3–54.6)	60.9 (53.4–68.4)	17.4 (4.3–30.5)	60.4 (51.5–69.3)

The prevalence estimates of tau positivity in the temporal cortex were generated from logistic GEE models stratified by syndrome diagnosis. Prevalence estimates in the total group were modeled using age as a determinant. Prevalence estimates according to Aβ status were modeled using age, Aβ status and an interaction between age and Aβ status. The analyses presented in this table are based on 7,394 CU participants (68.7 ± 11.1 years, 55.9% women), of whom 7,186 had Aβ status available (68.7 ± 11.1 years, 56.0% women), 2,177 participants with MCI (71.3 ± 8.8 years, 45.0% women), of whom 2,121 had Aβ status available (71.4 ± 8.8 years, 44.8% women) and 2,477 participants with dementia (69.9 ± 9.0 years, 50.9% women), of whom 2,252 had Aβ status available (69.9 ± 9.0 years, 51.2% women).

**Extended Data Fig. 3:**
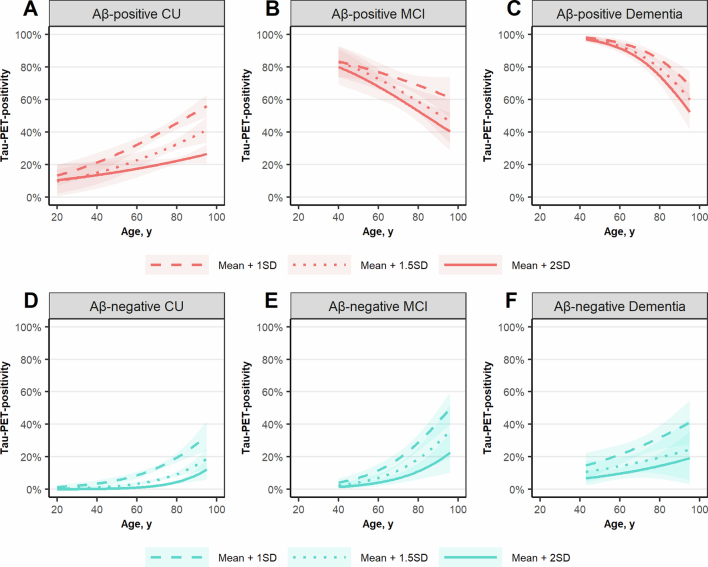
Prevalence estimates of Tau-PET positivity in the temporal cortex using lower thresholds. The plots depict the prevalence estimates of Tau-PET positivity in the temporal cortex as a function of age, Aβ-status and an interaction between age and Aβ-status in CU (panels **a** and **d**, *n* = 7,186), MCI (panels **b** and **e**, *n* = 2,121) and dementia (panels **c** and **f**, *n* = 2,252). Models were stratified by syndrome diagnoses. Tau-PET-positivity was defined using three different thresholds for Tau-PET positivity (that is, the mean + 1, 1.5 or 2 s.d. in Aβ-negative CU individuals). The *y* axes reflect estimated probabilities of tau-PET positivity (prevalence estimates) from logistic GEEs. Shaded areas indicates the 95% confidence intervals. Aβ = Amyloid-beta; CU = Cognitively unimpaired; MCI, mild cognitive impairment; PET = Positron emission tomography.

The same analyses presented above but now using cohort-specific thresholds of mean + 1 s.d. and 1.5 s.d. in Aβ-negative CU individuals yielded largely similar results (Extended Data Fig. 3), as well as analyses in which tau PET thresholds derived from tracer-specific Gaussian mixture modeling (instead of a cohort-specific mean + 2 s.d. in the Aβ-negative CU individual threshold used in the primary analyses; Supplementary Fig. 4). The observed tau PET-positivity prevalence by age, diagnosis and Aβ status is presented in Supplementary Table 3, to allow comparison against the estimated prevalence presented in Table 2.

### Tau positivity by age and *APOE* ε4 status in CU individuals

**Fig. 2:**
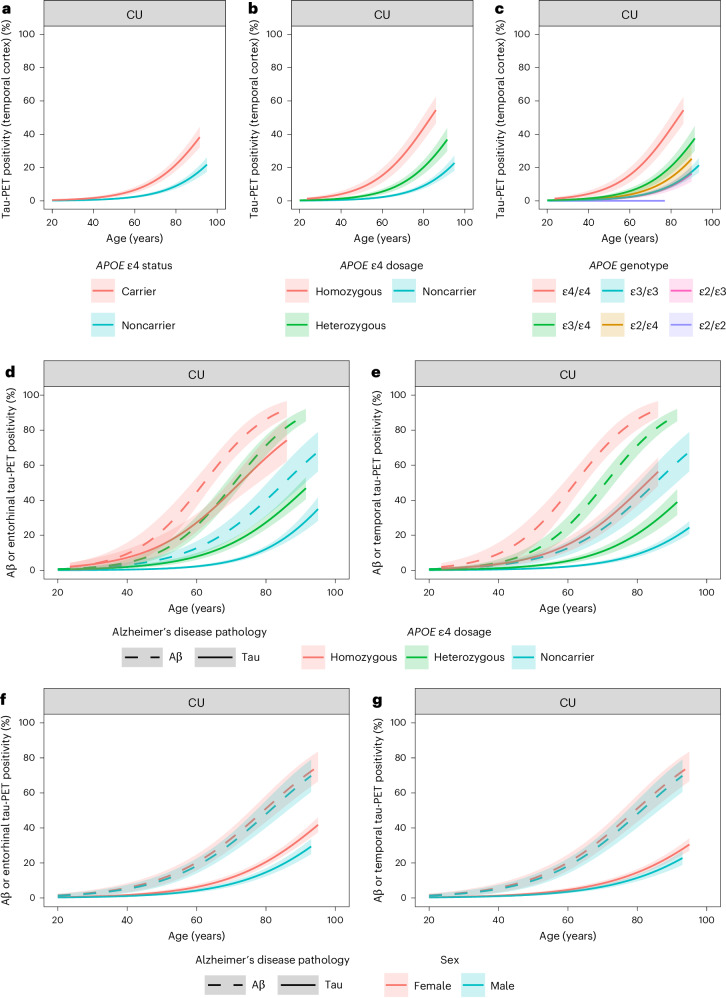
Prevalence estimates of tau PET and Aβ positivity by age, *APOE* and sex in CU individuals. **a**–**c**, The models including age and *APOE* ε4 status (**a**, *n* = 6,476), *APOE* ε4 dosage (**b**, *n* = 6,288) or *APOE* genotype (**c**, *n* = 5,963). **d**,**e**, The models including age and *APOE* ε4 dosage, with an additional interaction term between age and *APOE* ε4 dosage in the model estimating the prevalence of Aβ positivity (*n* = 6,184). **f**,**g**, The models including age and sex (*n* = 7,173). For the models presented in **d**–**g**, we included only individuals who had both Aβ status and entorhinal tau PET status and/or temporal cortex tau PET status available. Separate models were performed for estimating the prevalence of Aβ positivity and tau positivity. Note that **d** and **f** depict Aβ positivity or tau PET positivity in the entorhinal cortex, whereas **e** and **g** depict Aβ positivity or tau PET positivity in the temporal cortex. In **a**–**c**, the *y* axes reflect estimated probabilities of tau PET positivity (prevalence estimates) generated from GEEs. In **d**–**g**, the *y* axes reflect estimated probabilities of Aβ positivity or tau PET positivity (prevalence estimates) generated from GEEs. Shaded areas indicate the 95% CIs. In **c**, none of the participants with *APOE* ε2/ε2 were tau positive, hence no 95% CI was provided for this group.

In CU individuals, *APOE* ε4 status (*APOE* ε4^+^ versus *APOE* ε4^−^, *β* = 1.04, *P* < 0.001; Fig. 2a and Extended Data Table 5) and the number of *APOE* ε4 alleles (*APOE* ε4 homozygous versus *APOE* ε4 noncarrier, *β* = 2.06, *P* < 0.001, *APOE* ε4 heterozygous versus *APOE* ε4 noncarrier, *β* = 0.94, *P* < 0.001 and *APOE* ε4 homozygous versus *APOE* ε4 heterozygous, *β* = 1.12, *P* < 0.001; Fig. 2b and Supplementary Table 4) were associated with a higher estimated prevalence of tau positivity in the temporal cortex. At the median age of 71 years, the prevalence estimates of tau positivity in the temporal cortex were higher in *APOE* ε4/ε4 compared with all other genotypes (mean difference ε4/ε4 versus ε2/ε3: 24.1% (95% CI 15.5–32.6%); ε4/ε4 versus ε2/ε4: 20.9% (95% CI 10.6–31.2%); ε4/ε4 versus ε3/ε3: 23.7% (95% CI 15.3–32.2%); and ε4/ε4 versus ε3/ε4: 16.8% (95% CI 9.4–24.2%); all *P* < 0.001) and higher in ε3/ε4 compared with ε2/ε3 and ε3/ε3 genotypes (mean difference ε3/ε4 versus ε2/ε3: 7.3% (95% CI 3.1–11.5%), *P* < 0.001; ε3/ε4 versus ε3/ε3: 6.9% (95% CI 2.8–11.0%); *P* < 0.001; Fig. 2c and Supplementary Table 5). No significant differences were found between the other genotypes and none of the 22 CU participants with an ε2/ε2 genotype were tau positive in any of the ROIs.

### Aβ and tau positivity by age and *APOE* ε4 in CU individuals

Next, we aimed to estimate the timing of biomarker positivity for both Aβ and tau pathology as a function of age and *APOE* status or genotype in CU individuals to capture the earliest stages of AD pathophysiology. Figure 2d,e and Extended Data Table 6 illustrate that an increasing *APOE* ε4 dose is associated with both Aβ and tau positivity occurring at a substantially younger age. The estimated age for 10% Aβ-positivity prevalence is 40.5 years in *APOE* ε4/ε4 carriers, 49.0 years in *APOE* ε3/ε4 carriers and 56.5 years in *APOE* ε4 noncarriers. The estimated age for 10% tau-positivity prevalence in the entorhinal cortex is 44.5 years in *APOE* ε4/ε4 carriers, 63.5 years in *APOE* ε3/ε4 carriers and 77.5 years in *APOE* ε4 noncarriers (Fig. 2d). The estimated age for 10% tau-positivity prevalence in the temporal cortex is 54.5 years in *APOE* ε4/ε4 carriers, 69.0 years in *APOE* ε3/ε4 carriers and 81.0 years in *APOE* ε4 noncarriers (Fig. 2e). These results imply that, at a group level, *APOE* ε4/ε4 CU individuals become tau positive in the entorhinal cortex at a younger age than *APOE* ε4 noncarriers become Aβ positive (Fig. 2d), whereas the prevalence curves for tau positivity in the temporal cortex in *APOE* ε4/ε4 carriers versus Aβ positivity in *APOE* ε4 noncarriers largely overlap (Fig. 2e).

### Aβ and tau positivity by age and sex in CU individuals

In CU individuals, female sex was associated with a higher estimated prevalence of Aβ positivity (*β* = 0.13, *P* = 0.005), tau positivity in the entorhinal cortex (*β* = 0.41; *P* < 0.001) and tau positivity in the temporal cortex (*β* = 0.27, *P* < 0.001; Fig. 2f,g) compared with male sex. The estimated age for 10% Aβ-positivity prevalence is 48.5 years for women and 50.0 years for men, the estimated age for 10% tau positivity in the entorhinal cortex is 67.5 years for women and 73.5 years for men (Fig. 2f) and the estimated age for 10% tau positivity in the temporal cortex is 73.0 years for women and 77.5 years for men (Fig. 2g and Extended Data Table 7). These results imply that, at a group level, CU women become Aβ positive and tau positive at a younger age than CU men.

### Tau positivity by age, APOE ε4 status and Aβ status

**Fig. 3:**
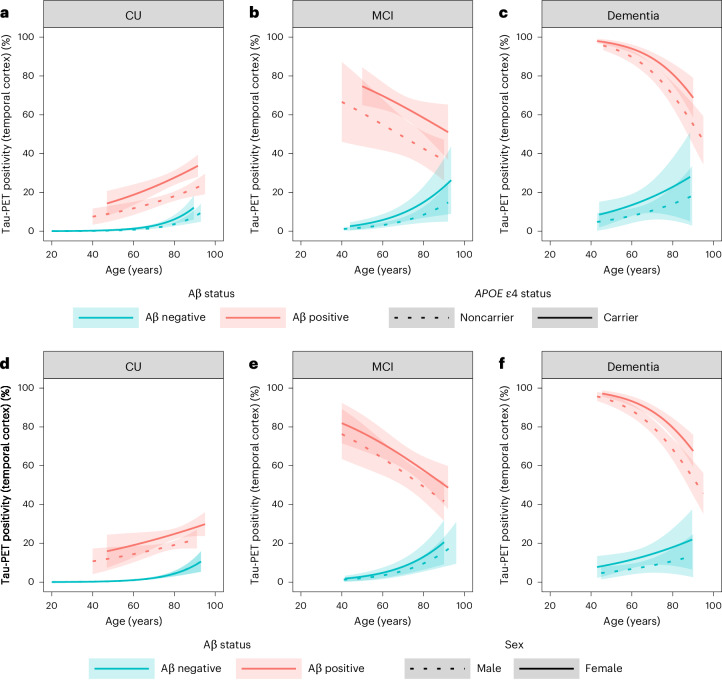
Tau PET positivity in association with age, Aβ status, *APOE* ε4 status and sex. **a**–**c**, Models including age, Aβ status, *APOE* ε4 status and an interaction between age and Aβ status for CU (**a**), MCI (**b**) and dementia (**c**). **d**,**e**, Models including age, Aβ status, sex and interaction terms between age and Aβ status (**d**) and between sex and Aβ status (**e**). **f**, Models including age, Aβ status, sex and an interaction term between age and Aβ status. Models were stratified for CU (**a** (*n* = 6,384) and **d** (*n* = 7,185)), MCI (**b** (*n* = 1,823) and **e** (*n* = 2,121)) and dementia (**c** (*n* = 1,869) and **f** (*n* = 2,252)) participants. The *y* axes reflect estimated probabilities of tau PET positivity in the temporal cortex (prevalence estimates) from GEEs. Shaded areas indicate the 95% CIs. Note that in **d**, the estimated probabilities and 95% CIs for the Aβ-negative men and Aβ-negative women are fully overlapping.

Given that the age effect on tau positivity was strongly modulated by Aβ status (for example, positive associations in Aβ-negative individuals with MCI or dementia versus negative associations in Aβ-positive individuals with MCI or dementia), we next modeled age, Aβ status and *APOE* ε4 status simultaneously. In models adjusting for age and Aβ pathology, *APOE* ε4 carriership was associated with a higher prevalence of tau positivity in the temporal cortex in all diagnostic groups (*β* = 0.55 for CU, *β* = 0.64 for MCI and *β* = 0.59 for dementia; all *P* < 0.001; Fig. 3a,c and Supplementary Table 6). For example, at the median age of 71 years, the prevalence estimates of tau positivity in the temporal cortex were higher in Aβ-positive *APOE* ε4 carriers compared with Aβ-positive *APOE* ε4 noncarriers in CU individuals (mean difference 8.4% (95% CI 3.0–13.8%), *P* < 0.001), individuals with MCI (mean difference 15.6% (95% CI 4.7–26.5%), *P* = 0.001) and those with dementia (mean difference 7.5% (95% CI 3.1–11.9%), *P* < 0.001).

### Tau positivity according to age, sex and Aβ status

In line with the previous section, we simultaneously modeled age, Aβ status and sex as predictors of tau positivity. There was a significant interaction between sex and Aβ status on the prevalence of tau positivity in the temporal cortex for CU (*β* = 0.34, *P* = 0.02) participants, indicating that, in the presence of Aβ pathology, CU women showed a higher prevalence of tau positivity than men (Fig. 3d,e and Supplementary Table 7). The interaction between sex and Aβ status on the prevalence of tau positivity was not significant in the MCI and dementia groups, but there were significant main effects of sex on tau positivity in the MCI (*β* = 0.34, *P* < 0.001) and dementia (*β* = 0.59, *P* < 0.001) groups (Fig. 3f and Supplementary Table 7). At the median age of 71 years, the prevalence estimates of tau positivity in the temporal cortex were higher in Aβ-positive women compared with Aβ-positive CU men (mean difference 5.2% (95% CI 1.7–8.8%), *P* = 0.001), MCI (mean difference 8.2% (95% CI 2.1–14.3%), *P* = 0.003) and dementia (mean difference 8.1% (95% CI 4.6–11.6%), *P* < 0.001).

### Tau positivity using tau PET versus postmortem examination

**Fig. 4:**
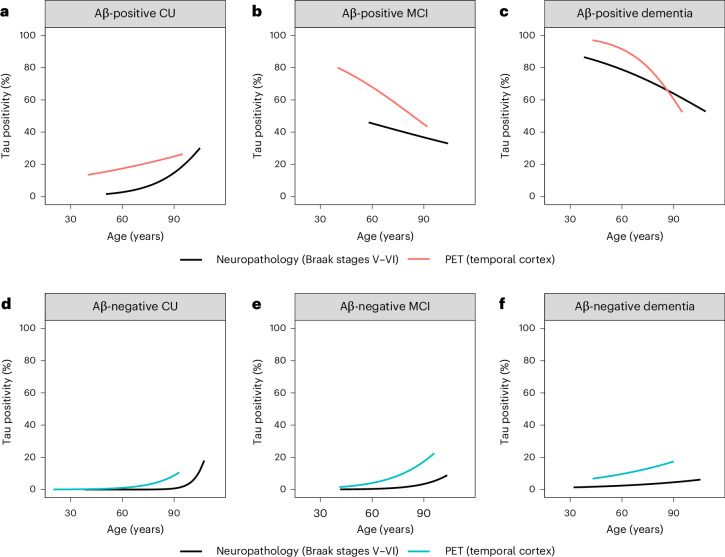
Prevalence of tau positivity on PET (temporal cortex) versus neuropathological examination (Braak V–VI). **a**–**f**, Tau positivity on PET or neuropathology in Aβ-positive (**a**) and Aβ-negative (**d**) CU individuals and Aβ-positive (**b**) and Aβ-negative (**e**) participants with MCI and Aβ-positive (**c**) and Aβ-negative dementia (**f**), modeled using age, Aβ status and an interaction between age and Aβ status as determinants. The models were stratified by syndrome diagnosis. The *y* axes reflect estimated probabilities of tau positivity on PET (temporal cortex) or neuropathology (Braak V–VI) (prevalence estimates) generated from GEEs. Prevalence estimates for PET are based on 7,186 CU, 2,121 MCI and 2,252 dementia participants. Prevalence estimates for neuropathology are based on 1,026 CU, 661 MCI and 3,385 dementia participants.

Although neuropathological studies have shown that the vast majority of older individuals harbor some degree of tau tangle pathology in the temporal cortex, antemortem tau PET versus postmortem comparisons indicated that tau PET scans typically become positive when tau tangle pathology is observed in the Braak V–VI regions^18,24–28^. Although prevalence estimates of tau positivity were generally higher when assessed using tau PET (temporal cortex) compared with neuropathology, we found comparable effects of age and Aβ status on tau positivity in autopsy versus PET datasets (Fig. 4). In line with the tau PET results, from age 60 years to age 80 years the estimated prevalence of neuropathologically defined Braak stages V–VI increased from 0.0% (95% CI 0.0–0.0%) to 0.1% (95% CI 0.0–0.3%) among Aβ-negative CU participants and from 2.6% (95% CI 0.0–7.9%) to 8.6% (95% CI 0.2–16.9%) among Aβ-positive CU participants (Fig. 4a,d and Supplementary Table 8). Among Aβ-negative participants with MCI and dementia, from age 60 years to 80 years, the estimated prevalence of Braak stages V–VI increased from 0.4% (95% CI 0.1–0.6%) to 1.7% (95% CI 1.3–2.1%) in MCI and from 2.4% (95% CI 0.0–9.5%) to 3.7% (95% CI 0.0–8.5%) in dementia (Fig. 4e,f and Supplementary Table 8). In contrast, among Aβ-positive participants with MCI and dementia, from age 60 years to 80 years, the estimated prevalence of Braak stages V–VI decreased from 45.6% (95% CI 0.0–98.0%) to 39.6% (95% CI 13.4–65.8%) in MCI and from 78.9% (95% CI 71.2–86.5%) to 69.4% (95% CI 61.1–77.8%) in dementia (Fig. 4b,c and Supplementary Table 8). A sensitivity analysis comparing tau positivity in neuropathologically defined Braak stages V–VI with tau PET positivity in a whole-brain ROI showed comparable results, although in Aβ-positive CU, the prevalence of tau PET positivity decreased with advancing age as opposed to postmortem Braak V–VI regions and tau PET positivity in the temporal cortex (Fig. 4a and Supplementary Fig. 5).

## Discussion

This large multicenter study aimed to estimate the prevalence of tau pathology as measured by PET as a function of Aβ status, age, *APOE* genotype and sex in CU individuals, individuals with MCI and individuals with dementia. Age and Aβ status showed the strongest associations with tau positivity. We found that age was positively associated with tau positivity in CU (irrespective of Aβ status) and showed a negative relationship with tau positivity in Aβ-positive individuals with MCI and Aβ-positive individuals with all-cause dementia. *APOE* ε4 carriership and female sex were associated with a higher prevalence of tau positivity across diagnostic groups. *APOE* ε4 carriership in CU individuals was associated with a lower age at onset of both Aβ positivity and tau positivity by decades in a dose-dependent fashion. Finally, the observed associations between age and Aβ status with tau pathology, as measured by PET, were validated in an independent autopsy dataset. Altogether, our study provides robust prevalence estimates of tau PET positivity across syndrome diagnoses and biomarker profiles, which can aid the interpretation of tau PET in the clinic and inform prevention studies and clinical trial designs.

One of the key findings of the present study is that carrying an *APOE* ε4 allele was associated with a lower age at onset of both Aβ positivity and tau positivity in CU individuals by decades in a dose-dependent fashion^5,6,29^. This shift is so pronounced that individuals with the *APOE* ε4/ε4 genotype exhibit tau PET positivity in the entorhinal cortex at a younger average age than *APOE* ε4 noncarriers become Aβ positive. To exemplify, the estimated ages at which 10% of CU *APOE* ε4/ε4 individuals show Aβ positivity (global) versus tau positivity (entorhinal cortex and temporal cortex) are 41, 45 and 55 years, respectively. In contrast, these estimated ages are 57, 78 and 81 years, respectively, for CU *APOE* ε4 noncarriers. Another key correlate of tau positivity in CU was Aβ status, because only 2.1% of Aβ-negative CU individuals were tau PET positive in the temporal cortex, whereas the prevalence was ~10-fold higher in Aβ-positive CU individuals. Also, the prevalence of entorhinal tau positivity was higher compared with the temporal tau positivity, which is in line with established neuropathological and PET-based staging schemes proposing this topography of tau progression^27,30,31^. Collectively, these data support a model where Aβ pathology triggers the spread of tau pathology from the medial temporal lobe to the neocortex, which is a critical harbinger of neurodegeneration and cognitive impairment in the near future^32,33^.

Age was also strongly associated with an increased prevalence of tau positivity in the entorhinal cortex and the temporal cortex in CU individuals, even among individuals who were Aβ negative. The latter can be explained by Aβ-independent tau accumulation (for example, primary age-related tauopathy (PART)), off-target binding of tau PET tracers (for example, to monoamine oxidase B, neuromelanin, iron accumulation and/or microhemorrhages, which all become more pronounced with advancing age), increased false-negative Aβ status and/or false-positive tau PET scans, partial volume effects resulting from atrophy or an atypical neurobiological phenotype (for example, a tau-first subtype)^34–39^. Among Aβ-positive CU individuals, contrary to entorhinal and temporal cortex tau positivity, tau positivity in the whole-brain ROI decreased in older age. This observation might be explained by a survival effect or a potentially increased susceptibility, in older participants, to the downstream neurotoxic effects of widespread tau pathology, for example, through less efficient compensatory neuronal mechanisms with age^40^. This reduced resilience might render older individuals more vulnerable to developing cognitive symptoms when tau aggregates are present in the neocortex, resulting in progression from CU to MCI or dementia at lower global levels of tau pathology. Consequently, a decrease of whole-brain tau positivity is observed in the CU group at older age. Longitudinal studies are essential to formally test the above proposed hypotheses.

At symptomatic AD stages (that is, Aβ-positive MCI and dementia), age was negatively associated with the prevalence of tau positivity in the entorhinal cortex, temporal cortex and whole-brain ROI. This finding is consistent with previous observations from both neuropathological and tau PET studies and this pattern has also been firmly established for Aβ pathology^29,41,42^. This observation can be explained in at least four distinct but not mutually exclusive ways. First, older individuals are more prone to co-occurring neuropathology like α-synuclein, TDP-43 or vascular injury^43^. According to the ‘double-hit’ hypothesis, even at lower (subthreshold) levels of tau pathology, this cumulative pathological burden may be sufficient to cause an MCI or dementia syndrome. Second, related to the above, decreased resilience to tau pathology with advanced age may lead to cognitive impairment at lower levels of tau pathological burden^44^. Third, individuals with advanced AD pathology in addition to substantial comorbid pathology are probably too cognitively impaired to participate in research studies and were thus potentially not included in our analyses. Fourth, misfolded tau proteins may spread or amplify faster in younger individuals with higher degrees of functional connectivity^45^. Also, younger individuals may be more susceptible to early deposition of tau pathology in hub network regions, which further accelerates the rate of tau accumulation^46^.

In CU individuals, we observed a lower age at onset for women relative to men for tau positivity in the entorhinal cortex and, more subtly, for Aβ positivity and tau positivity in the temporal cortex. Furthermore, in the CU group, we found that female sex was associated with a higher prevalence of temporal cortex tau positivity in the presence of Aβ pathology. This observation is in line with previous literature showing that clinical as well as biological AD are more common in women than in men: women exhibit a greater tau burden (particularly in the entorhinal cortex) at similar levels of Aβ pathology and Aβ-positive women show faster tau accumulation over time compared with Aβ-positive men^45,47,48^. Mechanisms relating to biological sex or social implications of gender could contribute to this difference, including (premature or early) menopause, late initiation of hormone therapy, differences in depression rates and educational attainment, as well as sex-specific innate and adaptive immune responses, synapse biology, mitochondrial functioning, neurotrophic factors and epigenetic alterations^49,50^.

An indirect comparison between tau positivity defined using PET in the temporal cortex versus neuropathological Braak stages V–VI showed similar associations with age and Aβ status across syndrome diagnostic groups. As a potential consequence of selecting advanced Braak stages as the primary neuropathological outcome measure in our study, the PET-based prevalence estimates were generally higher, particularly for Aβ-negative participants. This was still the case when we assessed a whole-brain ROI versus postmortem Braak stages V–VI. This may be related to differences in how the two modalities measure Aβ and tau pathology (for example, varying sensitivity and detection thresholds), differences in participants enrolled in PET versus autopsy studies or modality-specific measurement errors (for example, off-target binding in PET). There have been relatively few direct antemortem PET versus postmortem neuropathology comparison studies to date. Most of these showed a good correlation between tau PET signal and neuropathological Braak stages, and this correlation is further strengthened when, rather than the rather crude Braak staging, a more quantitative measure of postmortem tau pathology was used, such as the percentage of tissue stained by AT8 immunohistochemistry^18,25,51^. More multimodal studies are needed to better understand the overlap and differences between tau pathology as detected by PET versus at neuropathological examination, preferably assessed in the same individuals.

The main strength of this work is the large sample size (*N* = 12,048 for the PET sample and an additional *n* = 5,072 for postmortem validation) which allowed sufficient statistical power to provide robust prevalence estimates of tau PET positivity as a function of Aβ pathology and other individual risk factors for AD-type dementia such as age, *APOE* genotype and sex. Several limitations need to be considered when interpreting the present study. First, even though we include a global sample, generalizability is still limited because participants were, overall, highly educated (~14 years), mainly non-Hispanic white (79.5% of individuals had available data on race or ethnicity) and there were relatively few individuals aged >80 years, although this age range represents the largest segment of individuals with dementia in the community. Furthermore, data on race and/or ethnicity were available in only 54.6% of participants, which, combined with the overrepresentation of non-Hispanic white individuals, did not provide sufficient statistical power for conducting stratified analyses. Second, we pooled data from many cohorts. Although we used study-specific thresholds in the primary analyses and accounted for study effects within our statistical models, this may still have resulted in reduced internal validity as a result of differences in study designs. Third, owing to the absence of histopathological data in participants with tau PET (this was compared only in independent datasets), the present study lacked a gold standard. Fourth, we pooled data from four tau PET tracers that share similar properties but also show differences in tracer kinetics, selectivity and affinity, as well as differences in the degree and type of off-target binding patterns. Efforts are ongoing to harmonize tau PET data across tracers along common scales such as CenTauR^52^ or uniτ (that is, equivalent to the Centiloid approach for amyloid-PET^53^), which will improve future multicenter studies and trials that include tau PET. Fifth, there is no broad consensus on the most optimal way of operationalizing tau PET positivity quantitatively^54^. We acknowledge that several of our methodological decisions have impacted the reported prevalence estimates. In line with previous work^19^ we focused on AD-specific regions, which has potentially resulted in an underestimation of tau PET positivity in primary tauopathies characterized by differential tau patterns^55^. Also, we used the mean + 2 s.d. in Aβ-negative CU individuals aged >50 years as a threshold, whereas more liberal (for example, mean + 1.5 s.d.) or conservative (for example, mean + 2.5 s.d.) approaches could be considered for detecting early stage versus later-stage tau pathology^56^, respectively. Cohort-specific selection of reference regions, brain atlases and processing methods also all influence tau PET quantification. We have partially addressed these potential confounding factors by adjusting all statistical analyses for cohort and validating the main results using an alternative threshold method (that is, Gaussian mixture modeling or lower thresholds). However, some residual variability and imprecision probably remain. Sixth, although, to our knowledge, this is one of the largest tau PET studies to date, the sample size for some specific subgroup analyses was relatively small and resulted in wide CIs. In particular, the prevalence estimates associated with age and *APOE* genotype in MCI and dementia at the lower and higher age extremes should be interpreted with caution.

In conclusion, among people with and without cognitive impairment, the prevalence of tau pathology as determined by PET imaging was associated with Aβ status, age, sex and *APOE* genotype. Our findings support the clinical utility of tau PET for differential diagnosis and inform trial designs that utilize tau PET for participant selection and stratification. In terms of future directions, it will be important to (1) compare the tau PET prevalence estimates against biofluid (cerebrospinal fluid or plasma) markers of soluble tau pathology such as p-tau217 or MTBR-243 (refs. ^57,58^), (2) conduct a similar study with adjusted ROIs in other populations such as primary tauopathies (for example, globus pallidus in PSP^55^) or atypical variants of AD (for example, occipital cortex in posterior cortical atrophy^41^), (3) assess genetic effects beyond *APOE* genotype on tau PET prevalence^59^ and (4) repeat the current analyses once approaches of harmonization across different tau PET tracers are more advanced.

## Methods

Written informed consent was obtained from all participants or their designated caregiver and all data collection protocols were approved by each cohort’s respective institutional ethical review board. Data analysis protocols for this particular study were approved by the Ethics Committee of Lund University, Lund, Sweden, in accordance with the Declaration of Helsinki, and all methods were carried out in accordance with the approved guidelines.

### Data collection and operationalization

We searched the MEDLINE and Web of Science databases for tau PET studies published before 15 November 2023. The search terms used were ‘PET’ and ‘tau’ in combination with the four most widely used tau PET tracers to date (that is, ‘AV1451/flortaucipir/Tauvid’, ‘MK6240’, ‘RO948’ or ‘PI2620’). Based on titles and abstracts we identified 42 unique cohorts that had previously published tau PET data in peer-reviewed journals. These cohorts represented a mix of secondary and tertiary care research studies, population-based studies and the placebo arm of a clinical trial. We approached study contact people to request participant-level data: 38 cohorts accepted and 4 declined; 4 additional cohorts (that is, Barcelona Beta, UCL, Gothenburg University and the Chinese Preclinical Alzheimer’s disease Study) provided currently unpublished tau PET data, totaling participant-level data from 42 cohorts for analysis. Tau PET data were shared through transfer of raw PET images to be centrally processed at Lund University in line with previous procedures (16 cohorts, *n* = 4,296)^19^ or transfer of spreadsheets containing regional standardized uptake value (SUVR) data (26 cohorts, *n* = 7,752). In addition, data were shared regarding clinical diagnosis (42 cohorts), Aβ status (41 cohorts), Aβ modality (39 cohorts), PET (938 cohorts) and/or cerebrospinal fluid (CSF) (10 cohorts), age (42 cohorts), sex (42 cohorts), education (36 cohorts), race or ethnicity (21 cohorts), *APOE* ε4 status (38 cohorts), *APOE* genotype (35 cohorts) and Mini-Mental State Examination (MMSE) score (42 cohorts). Cohort-specific methods for defining Aβ status are presented in Supplementary Table 9. We excluded participants with missing tau PET SUVR in the temporal cortex (*n* = 8), missing syndrome diagnosis (*n* = 234) and genetic mutations associated with dementia (*n* = 8) and participants with MCI or dementia who were aged <40 years (*n* = 7).

### Participants

Informed consent was obtained from all participants or their assigned surrogate decision-makers and the institutional review boards for human research of the participating centers approved all studies. CU individuals performed cognitive testing within normal limits and did not exhibit any major psychiatric disorder^60^. MCI was defined according to published criteria^61,62^. These criteria include a decline in memory or another cognitive domain reported by the patient, informant or both, that is, objectively verified by neuropsychological testing, in combination with no or minimal impairment in activities of daily living and not meeting criteria for dementia. Patients with a syndromic dementia diagnosis met diagnostic criteria for AD-type dementia^63^ (*n* = 1,804) or non-AD neurodegenerative disorders including FTD (*n* = 162, that is, behavioral variant FTD and the semantic and nonfluent variants of primary progressive aphasia combined), PSP (*n* = 141), CBS (*n* = 101), DLB (*n* = 76), PDD (*n* = 39), VaD (*n* = 32) and dementia–not otherwise specified (NOS) (*n* = 122). Note that we reported results for ‘all-cause dementia’ (that is, all types of dementia combined) in the main text, whereas results for the specific dementia types are reported in Extended Data Fig. 1 and Supplementary Information. In addition, we repeated the main analyses presented in Figs. 1 and 3 specifically for individuals clinically diagnosed with AD-type dementia (Extended Data Fig. 2 and Extended Data Table 4). In addition, for an (indirect) comparison between tau-positivity rates derived from tau PET versus neuropathological examination, we included 5,072 participants from 3 autopsy cohorts (that is, the National Alzheimer’s Coordinating Center database (NACC, *n* = 1,638)^64^, the Religious Orders Studies and Rush Memory and Aging Project (ROSMAP, *n* = 1,941)^65^ and the Arizona Study of Aging and Neurodegenerative Disorders (AZSAND)/Brain and Body Donation Program (AZSAND/BBDP, *n* = 1,672)^66^). The combined autopsy dataset consisted of 1,026 CU individuals, 661 individuals with MCI and 3,385 with dementia (Extended Data Table 1). In line with previous work^29^, participants who met the Consortium to Establish a Registry for Alzheimer’s Disease criteria (CERAD)^67^ for definite, probable or possible AD (indicating the presence of moderate-to-frequent neuritic plaques) were considered Aβ positive. Based on previous results of antemortem tau PET versus postmortem examination in the same individuals, participants in Braak stage V–VI for neurofibrillary tangle pathology were considered tau positive^18,24–27^. We compared the prevalence of postmortem Braak stage V–VI against tau PET positivity in the temporal cortex (Fig. 4) and a whole-brain ROI (Supplementary Fig. 5). Participants with missing antemortem diagnosis, age, CERAD score or Braak stage were excluded from the autopsy dataset.

### Tau PET procedures

[^18^F]Flortaucipir was used in most patients (*n* = 6,480, 25 cohorts), followed by [^18^F]MK6240 (*n* = 3,156, 11 cohorts), [^18^F]RO948 (*n* = 1,984, 3 cohorts) and [^18^F]PI2620 (*n* = 428, 4 cohorts). Cohort-specific information on tau PET tracers, scanning procedures and data processing can be found in Supplementary Table 10. For the primary analysis, we focused on a composite temporal meta-ROI (referred to as ‘temporal cortex’ throughout the text for readability purposes), consisting of the entorhinal cortex, amygdala, parahippocampus, fusiform gyrus and inferior and middle temporal cortices^68^. In addition, we determined tau PET positivity in the entorhinal cortex (missing for *n* = 35) and in a whole-brain ROI (missing for *n* = 2; see Supplementary Table 11 for cohort-specific ROI compositions). Tau PET scans were dichotomized (positive or negative) using quantitative thresholds. For the primary analyses, we defined the cut-off based on cohort-specific thresholds calculated as the mean + 2 s.d. in Aβ-negative CU individuals aged >50 years from the same cohort (see Supplementary Table 12 for cohort-specific thresholds). In sensitivity analyses, we also showed the results when determining the threshold based on the mean + 1 s.d. and 1.5 s.d. in Aβ-negative CU individuals aged >50 years from the same cohort (Extended Data Fig. 3). Furthermore, we defined the cut-off based on tracer-specific Gaussian mixture modeling ([^18^F]flortaucipir: SUVR = 1.40; [^18^F]MK6240: SUVR = 1.43; [^18^F]RO948: SUVR = 1.41; [^18^F]PI2620: SUVR = 1.41 in the temporal cortex; see Supplementary Table 12 for tracer-specific thresholds for the entorhinal and whole-brain ROIs).

### Statistical analysis

Baseline characteristics were compared using analysis of variance (ANOVA) and Fisher’s exact tests, where appropriate. GEEs were used to estimate probabilities of tau PET positivity. GEEs were selected because they allowed the modeling of subject-level data from all studies simultaneously while accounting for the clustering of participants within studies. Furthermore, GEEs provided population-averaged estimates (that is, coefficients representing the average effect on tau PET positivity across the dataset population) as opposed to subject-specific estimates, where coefficients represented the effect on tau PET positivity for the average individual in the dataset. We assumed a logit link function for binary outcomes with an exchangeable correlation structure to account for within-study correlations related to, for example, site-specific PET scanners and study populations. All data were visually inspected and data distribution was assumed to be normal, but this was not formally tested. The main analyses were performed stratified for syndrome diagnosis and included Aβ status (±), age, sex and/or *APOE* ε4 status (±) as independent variables. Age was entered as a continuous measure centered at the median (that is, 71 years). We tested two-way and three-way interactions between variables and these terms were retained in the model if they appeared significant by Wald’s statistic (indicated in table footnotes and figure legends). We used estimated probabilities and 95% CIs from the GEE analyses in tables and figures. These GEE-estimated probabilities were compared with observed probabilities to determine the goodness of fit between GEE-estimated and actual data and these comparisons are presented in Supplementary Table 3. In addition, we modeled Aβ positivity and tau PET positivity as a function of age and *APOE* ε4 dose (that is, homozygous versus heterozygous versus noncarrier) and as a function of age and sex, and we compared the estimated tau-positivity prevalence as determined in tau PET versus postmortem datasets. The significance level was set at *α* = 0.05 and the analyses were performed using R v.4.2.1.

### Reporting summary

Further information on research design is available in the Nature Portfolio Reporting Summary linked to this article.

## Online content

Any methods, additional references, Nature Portfolio reporting summaries, source data, extended data, supplementary information, acknowledgements, peer review information; details of author contributions and competing interests; and statements of data and code availability are available at 10.1038/s41593-025-02000-6.

## Supplementary information

Supplementary Information Supplementary Figs. 1–3 and Tables 1–7.

Reporting Summary

## Figures and Tables

**Extended Data Table 1:**
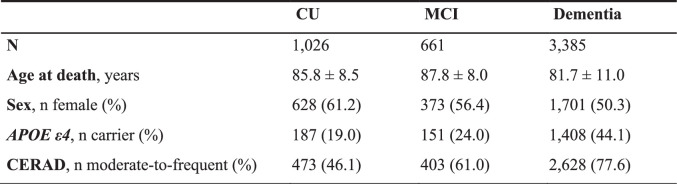
Participant characteristics of the autopsy cohorts

CERAD, Consortium to Establish a Registry for Alzheimer’s Disease criteria.

**Extended Data Table 2:**
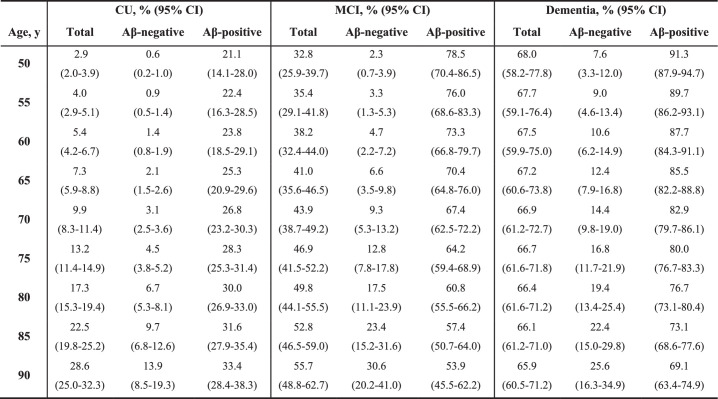
Prevalence estimates of tau-PET positivity in the entorhinal cortex according to age, Aβ and cognitive status

The prevalence estimates of tau positivity in the entorhinal cortex were generated using logistic GEE models stratified by syndrome diagnosis. Prevalence estimates in the total group were modeled using age as determinant. Prevalence estimates according to Aβ status were modeled using age, Aβ status and an interaction term between age and Aβ status and models were stratified by syndrome diagnosis. The analyses presented in this table are based on 7,381 CU participants (68.7 ± 11.1 years, 56.0% female). of whom 7,174 had Aβ status available (68.7 ± 11.1 years, 56.0% female), 2,173 participants with MCI (71.3 ± 8.8 years, 45.1% female). of wom 2,117 had Aβ status available (71.3 ± 8.8 years, 44.9% female) and 2,459 participants with dementia (69.9 ± 9.0 years, 50.6% female). of whom 2,234 had Aβ status available (69.9 ± 9.0 years, 51.2% female).

**Extended Data Table 3:**
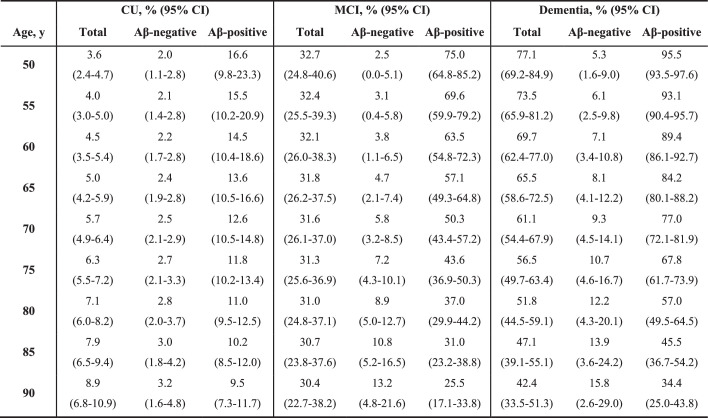
Prevalence estimates of tau-PET positivity in the whole-brain ROI according to age, Aβ and cognitive status

The prevalence estimates of tau positivity in the whole-brain ROI were generated using logistic GEE models stratified by syndrome diagnosis. Prevalence estimates in the total group were modeled using age as determinant. Prevalence estimates according to Aβ status were modelled using age, Aβ status and an interaction term between age and Aβ status, and models were stratified by syndrome diagnosis. The analyses presented in this table are based on 7,394 CU participants (68.7 ± 11.1 years, 55.9% female), of whom 7,186 had Aβ status available (68.7 ± 11.1 years, 56.0% female), 2,177 participants with MCI (71.3 ± 8.8 years, 45.0% female), of whom 2,121 had Aβ status available (71.4 ± 8.8 years, 44.8% female) and 2,475 participants with dementia (69.9 ± 9.0 years, 50.7% female), of whom 2,252 had Aβ status available (69.9 ± 9.0 years, 51.2% female).

**Extended Data Table 4:**
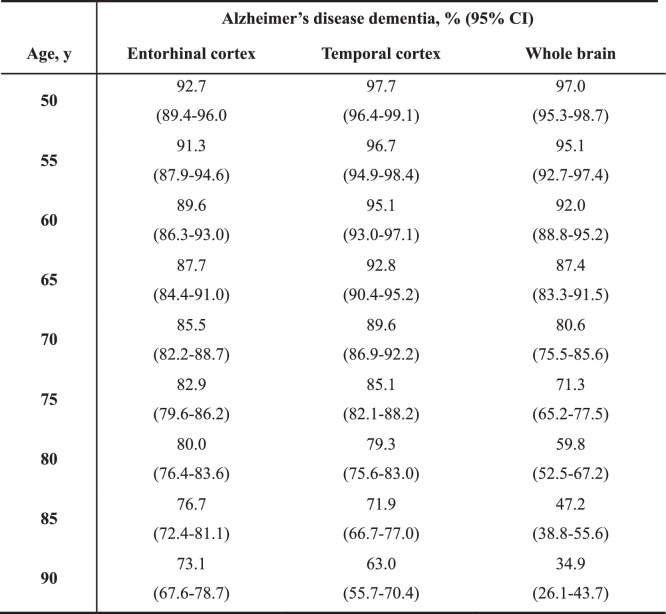
Prevalence estimates of tau-PET-positivity in amyloid-positive AD-type dementia

The prevalence estimates of tau-PET positivity in the entorhinal cortex, temporal cortex,and whole brain were generated using logistic GEE models according to age and Aβ in participants clinically diagnosed with AD-type dementia *(n* = 1,696).

**Extended Data Table 5:**
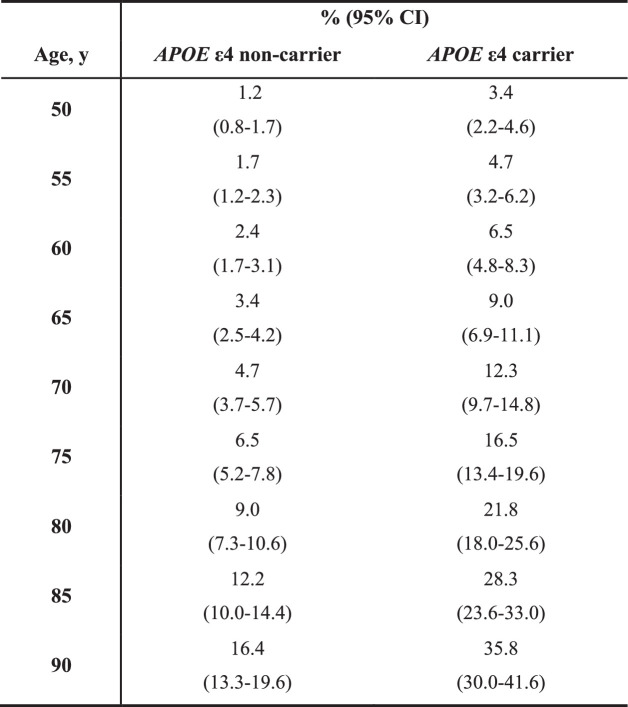
Age by *APOE* ε4 status in CU individuals

The prevalence estimates of tau positivity in the temporal cortex were generated from logistic GEE models including age and *APOE* ε4 status (*n* = 6,476).

**Extended Data Table 6:**
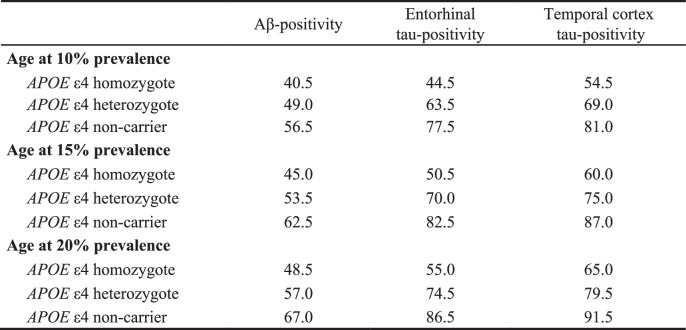
Aβ and tau positivity by age and *APOE* in CU individuals

The prevalence estimates of Aβ and tau positivity were generated using logistic GEE models including age and *APOE* ε4 dosage, and only included individuals that had Aβ status, entorhinal tau-PET status and temporal cortex tau-PET status available (*n* = 6,184). Separate models were performed for estimating the prevalence of Aβ positivity and tau positivity. Models estimating the prevalence of Aβ positivity and entorhinal tau positivity additionally included an interaction term between age and *APOE* ε4 dosage.

**Extended Data Table 7:**
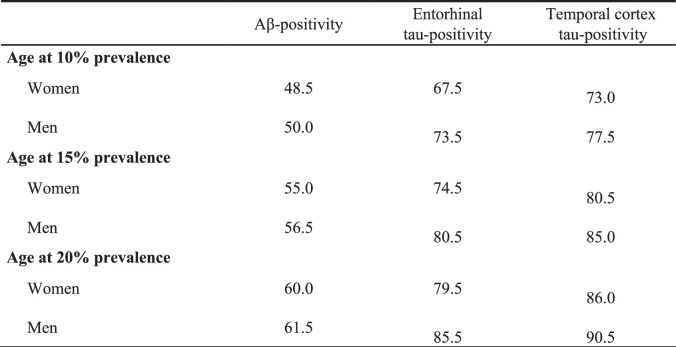
Aβ and tau positivity by age and sex in CU individuals

The prevalence estimates of Aβ and tau positivity were generated using logistic GEE models including age and sex and models only included individuals that had Aβ status, entorhinal tau-PET status and temporal cortex tau-PET status available (*n* = 7,173). Separate models were performed for estimating the prevalence of Aβ positivity and tau positivity.

## Data Availability

As a result of the multicenter design of the study, individual participant data from each cohort will have to be made available through the principal investigators of the respective cohorts. Generally, anonymized data can be shared by request from qualified academic investigators for the purpose of replicating procedures and results presented in the Article, if the data transfer is in agreement with the data protection regulation at the institution and approved by the local ethics review board.

## Code availability

The codes used for data collection in our study were implemented in R v.4.2.1 and can be requested from the corresponding authors (R.O. or O.H.). The codes used for data analysis were implemented in R v.4.2.1 and are available via GitHub at https://github.com/OssenKoppeLab.
