# Supplementary material for: Tau PET positivity in individuals with and without cognitive impairment varies with age, amyloid-β status, APOE genotype and sex
Source: Nat Neurosci. 2025 Jul 16;28(8):1610–21. doi: 10.1038/s41593-025-02000-6 (PMC12321570; doi:10.1038/s41593-025-02000-6)
Supplement: Supplementary file 1 — Supplementary Figs. 1–3 and Tables 1–7. [file 41593_2025_2000_MOESM1_ESM.pdf]

nature neuroscience

Supplementary information https://doi.org/10.1038/s41593-025-02000-6

Tau PET positivity in individuals with and without cognitive impairment varies with age, amyloid-β status, *APOE* genotype and sex

In the format provided by the authors and unedited

SUPPLEMENTARY CONTENT

Table/Figure	Title	Page
Table 1	Participant characteristics stratified by Aβ-status	2
Figure 1	Observed tau-positivity in the entorhinal cortex across diagnostic groups	3
Figure 2	Observed tau-positivity in the whole brain across diagnostic groups	4
Table 2	Observed tau positivity prevalence in (Aβ+) EOAD vs LOAD	5
Figure 3	Proportion of Tau-PET positivity across ROIs in (Aβ+) EOAD vs LOAD	6
Figure 4	Prevalence estimates of Tau-PET positivity according to age, Aβ and cognitive status using thresholds derived with Gaussian mixture models	7
Table 3	Observed prevalence of Tau-PET positivity in the temporal cortex according to age, Aβ and cognitive status	8
Table 4	Age by *APOE* ε4-dosage in cognitively unimpaired individuals	9
Table 5	Age by *APOE* ε4-genotype in cognitively unimpaired individuals	10
Table 6	Tau-PET-positivity in association with age, Aβ- and *APOE* ε4-status	11
Table 7	Tau-PET-positivity in association with age, Aβ-status and sex	12
Table 8	Prevalence estimates of tau-positivity in autopsy cohorts	13
Figure 5	Prevalence of Tau-positivity on PET (whole-brain ROI) vs neuropathological examination (Braak V-VI)	14
Table 9	Cohort-specific Ab-status information	15
Table 10	Cohort-specific Tau-PET information	17
Table 11	Cohort-specific Tau-PET ROI compositions	19
Table 12	Cohort- and tracer-specific Tau-PET thresholds	21
Figure 6	Cohort-specific histograms of Tau PET SUVR's in the temporal cortex in Aβ- CU individuals	23

Supplementary Table 1. Participant characteristics stratified by Aβ-status

	CU		MCI		Dementia	
	**Aβ-negative**	**Aβ-positive**	**Aβ-negative**	**Aβ-positive**	**Aβ-negative**	**Aβ-positive**
**N**	4,968	2,218	863	1,258	522	1,730
**Age, years**	66.4 ± 11.8	73.8 ± 7.4	69.8 ± 9.0	72.5 ± 8.4	69.3 ± 8.7	70.0 ± 9.1
**Sex, n female (%)**	2,777 (55.9)	1,249 (56.3)	352 (40.8)	599 (47.6)	232 (44.4)	921 (53.2)
***APOE ε4* status, n carrier (%)**	1,229 (28.0)	1,064 (53.3)	181 (24.2)	686 (63.8)	85 (23.8)	979 (64.7)
**Amyloid-β modality, n PET (%)**	4,525 (95.3)	2,059 (95.1)	774 (91.0)	1,118 (89.8)	351 (67.2)	1,300 (75.2)
**Education, years**	14.6 ± 3.7	14.8 ± 3.5	13.2 ± 4.3	13.6 ± 4.3	12.6 ± 4.5	13.2 ± 4.1
**MMSE**	28.8 ± 1.8	28.6 ± 1.4	27.3 ± 2.1	26.3 ± 2.6	23.4 ± 4.9	20.1 ± 6.0
**Race/ethnicity, n self-reported (% of total)**						
Non-hispanic white	2,422 (76.5)	1,304 (89.9)	345 (76.8)	521 (84.3)	93 (66.4)	506 (74.1)
Black or African American	228 (7.2)	54 (3.7)	24 (5.3)	23 (3.7)	5 (3.6)	19 (2.8)
Hispanic or Latino	288 (9.1)	26 (1.8)	13 (2.9)	13 (2.1)	4 (2.9)	9 (1.3)
Asian	211 (6.7)	59 (4.1)	67 (14.9)	57 (9.2)	38 (27.1)	143 (20.9)
American Indian or Alaskan native	7 (0.2)	2 (0.1)	0 (0.0)	0 (0.0)	0 (0.0)	2 (0.3)
Native Hawaiian or other Pacific islander	1 (0.0)	0 (0.0)	0 (0.0)	0 (0.0)	0 (0.0)	0 (0.0)
More than one	9 (0.3)	4 (0.3)	0 (0.0)	3 (0.5)	0 (0.0)	3 (0.4)
Other	2 (0.1)	2 (0.1)	0 (0.0)	1 (0.2)	0 (0.0)	1 (0.1)
**Tau-PET tracer, n (%)**						
$[^{18}F]$flortaucipir	2,523 (50.8)	1,512 (68.2)	451 (52.3)	641 (51.0)	226 (43.3)	957 (55.3)
$[^{18}F]$MK6240	1,599 (32.2)	399 (18.0)	212 (24.6)	361 (28.7)	90 (17.2)	360 (20.8)
$[^{18}F]$RO948	789 (15.9)	275 (12.4)	196 (22.7)	233 (18.5)	103 (19.7)	323 (18.7)
$[^{18}F]$PI2620	57 (1.1)	32 (1.4)	4 (0.5)	23 (1.8)	103 (19.7)	90 (5.2)

Shown are mean ± standard deviations unless specified otherwise. Sex was missing for 1 participant (0.0%), APOE ε4 status for 1483 participants (12.8%), Aβ modality for 300 participants (2.6%), education for 882 participants (7.6%), and MMSE for 663 participants (5.7%). Amyloid-β modality reflects the modality on which Aβ status was defined and could include either PET or CSF (see **Extended-Data Table-14**). Aβ = Amyloid-beta; CU = Cognitively unimpaired; MCI = Mild cognitive impairment; MMSE = Mini-mental state examination; PET = Positron emission tomography

Supplementary Figure 1. Observed tau-positivity in the entorhinal cortex across diagnostic groups

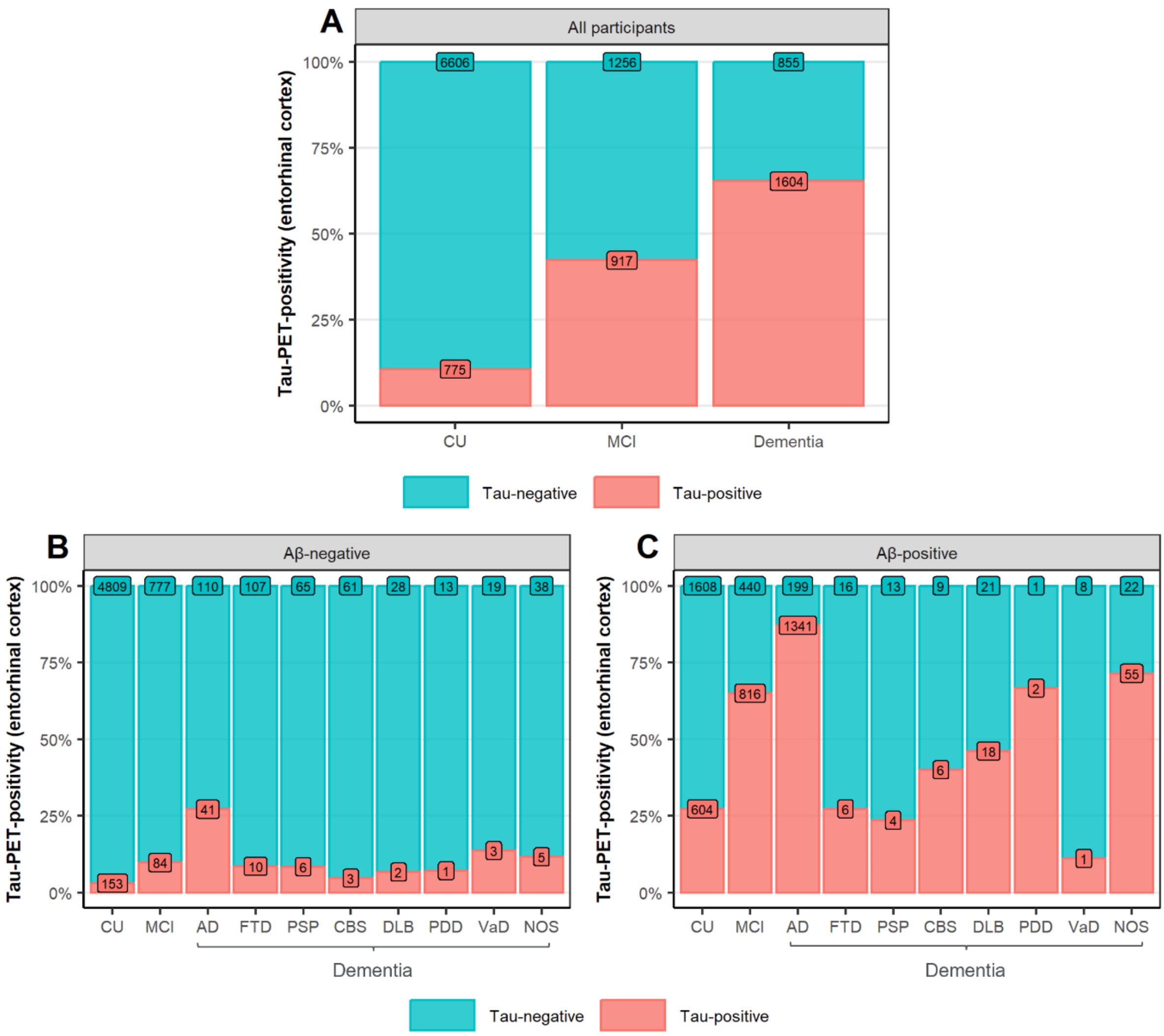

Plots show the observed rates of Tau-PET positivity in the entorhinal cortex by (syndromic and clinical) diagnosis for all participants (panel A, n=1,2013) or stratified by Aβ-status (panels B [n=6,335] and C [n=5,190]). Aβ = Amyloid-beta; AD = Alzheimer's disease, CBS = Corticobasal syndrome, DLB = Dementia with Lewy bodies, FTD = Frontotemporal dementia, MCI = Mild cognitive impairment, NOS = Not otherwise specified, PDD = Parkinson's disease dementia, PSP = Progressive supranuclear palsy, VaD = Vascular dementia.

Supplementary Figure 2. Observed tau-positivity in the whole brain across diagnostic groups

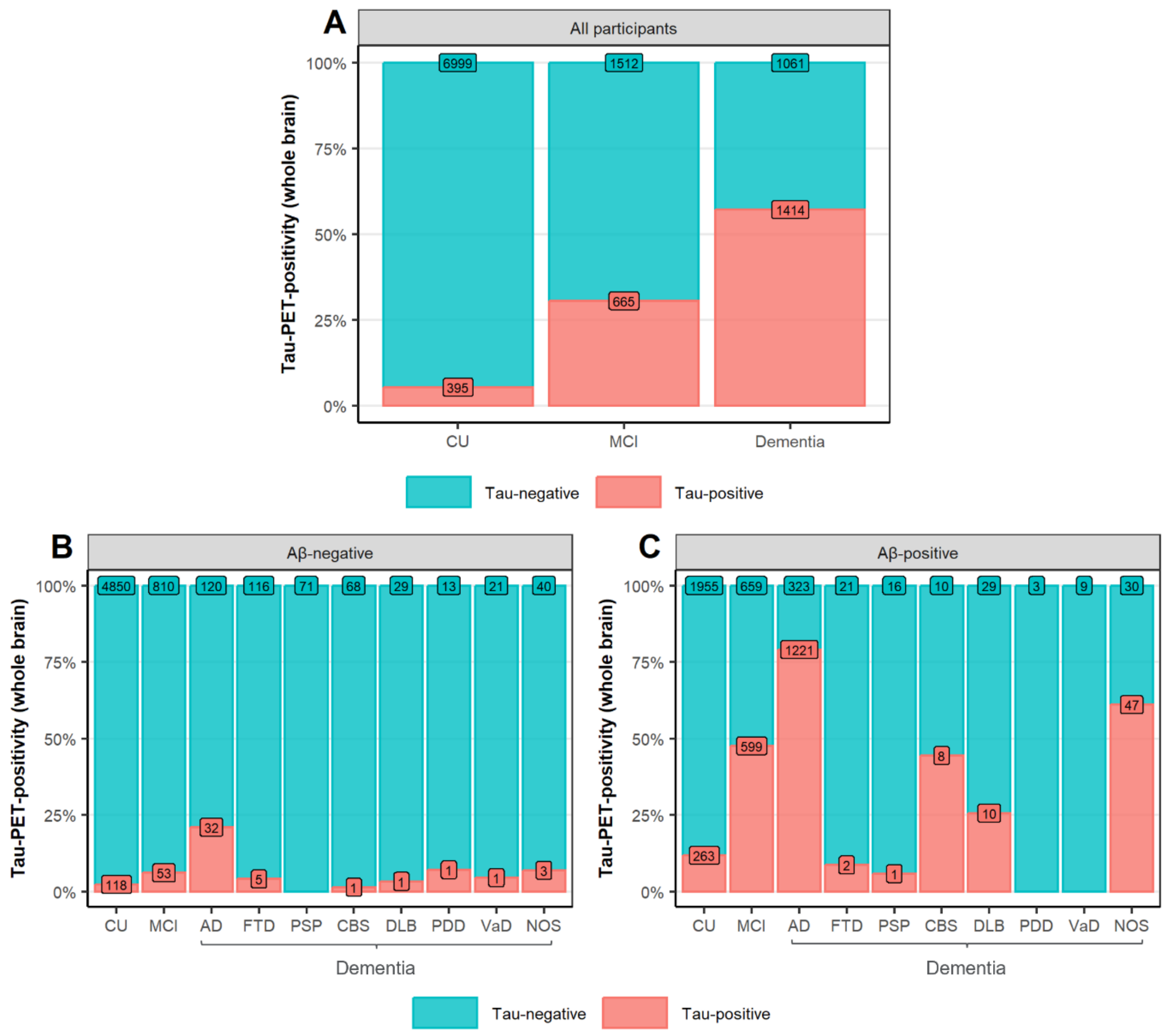

Plots show the observed rates of Tau-PET positivity in the whole-brain region-of-interest by (syndromic and clinical) diagnosis for all participants (panel A, n=1,2046) or stratified by Aβ-status (panels B [n=6,353] and C [n=5,206]). Aβ = Amyloid-beta; AD = Alzheimer's disease, CBS = Corticobasal syndrome, DLB = Dementia with Lewy bodies, FTD = Frontotemporal dementia, MCI = Mild cognitive impairment, NOS = Not otherwise specified, PDD = Parkinson's disease dementia, PSP = Progressive supranuclear palsy, VaD = Vascular dementia.

Supplementary Table 2. Observed tau positivity prevalence across ROIs in (Aβ+) early- vs late-onset Alzheimer's disease dementia

	Early-onset AD		Late-onset AD	
	Positive / Total	% Positive	Positive / Total	% Positive
Entorhinal cortex Tau PET	518/568	91.2	915/1162	78.7
Temporal cortex Tau PET	542/568	95.4	903/1162	77.7
Whole Brain Tau PET	535/568	94.2	754/1162	64.9
Braak V-VI post-mortem	171/189	90.5	1630/2439	66.8

EOAD=Early-onset Alzheimer's disease (<66 years at the time of PET), LOAD=Late-onset Alzheimer's disease (>65 years at the time of PET).

Supplementary Figure 3. The proportion of Tau-PET positivity across ROIs in (Aβ+) early- vs late-onset Alzheimer's disease dementia

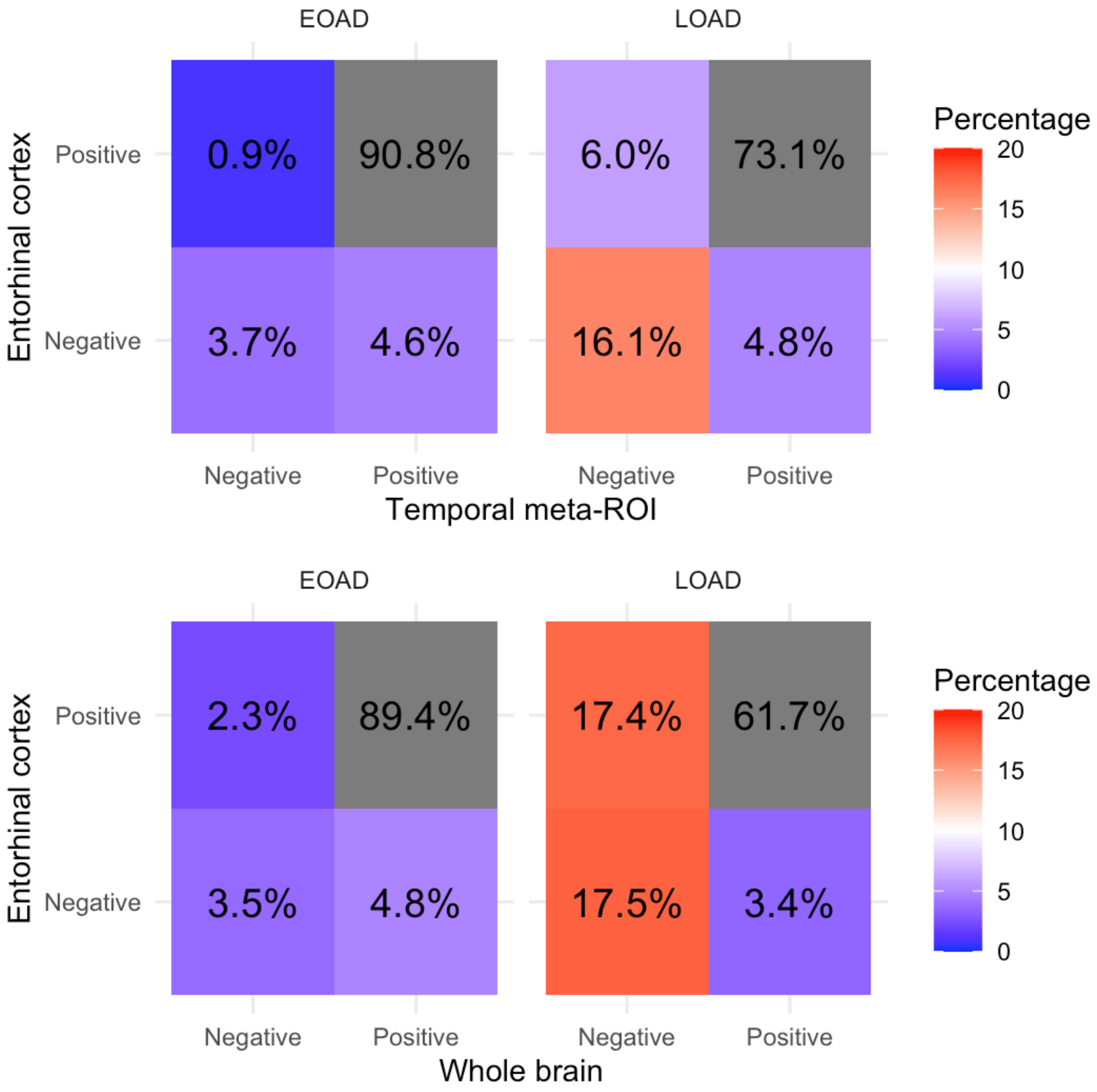

EOAD=Early-onset Alzheimer's disease (<66 years at the time of PET), LOAD=Late-onset Alzheimer's disease (>65 years at the time of PET).

Supplementary Figure 4. Prevalence estimates of Tau-PET positivity according to age, Aβ and cognitive status using thresholds derived with Gaussian Mixture Modeling

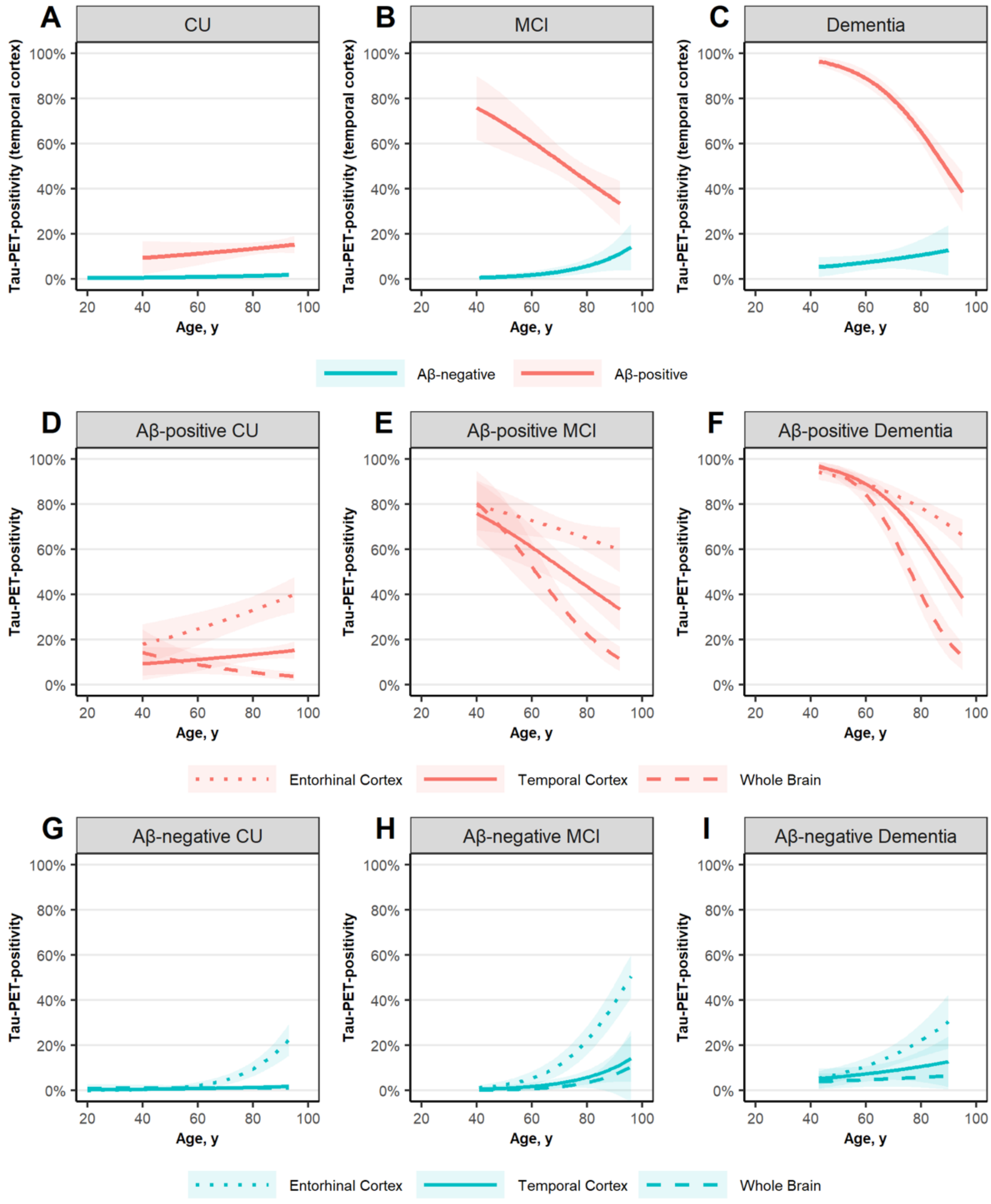

Tau-PET positivity in CU (panels A, D and G), MCI (panels B, E and H) and dementia (panels C, F and I) was modelled using age, Aβ-status and an interaction term between age and Aβ-status as determinant. Models were stratified by syndrome diagnosis. Tau-PET positivity was assessed in the temporal cortex (shown in all panels) as well as in the entorhinal cortex and whole brain (shown in panels D-I). The interaction between age and Aβ status was not significant for the whole-brain ROI in CU individuals and was, therefore (only) removed from this model. The y-axes reflect estimated probabilities of Tau-PET-positivity (prevalence estimates) from generalized estimating equations. The figure includes 7,186 CU, 2,121 MCI and 2,252 dementia participants for Tau-PET-positivity in the temporal cortex and whole brain region; and 7,174 CU, 2,117 MCI and 2,234 dementia participants for Tau-PET-positivity in the entorhinal cortex. Shading areas indicate the 95% confidence intervals. Aβ = Amyloid-beta; CU = Cognitively unimpaired; MCI = Mild cognitive impairment; PET = Positron emission tomography.

Supplementary Table 3. Observed prevalence of Tau-PET positivity in the temporal cortex according to age, Aβ and cognitive status

	CU, % (n positive / n total)			MCI, % (n positive / n total)			Dementia, % (n positive / n total)		
**Age, y**	**Total**	**Aβ-negative**	**Aβ-positive**	**Total**	**Aβ-negative**	**Aβ-positive**	**Total**	**Aβ-negative**	**Aβ-positive**
**47.5-52.4**	1.7 (3/182)	0.0 (0/167)	25.0 (2/8)	28.6 (8/28)	0.0 (0/14)	61.5 (8/13)	77.3 (34/44)	11.1 (1/9)	100.0 (31/31)
**52.5-57.4**	0.3 (1/373)	0.3 (1/339)	0.0 (0/19)	37.3 (38/102)	0.0 (0/50)	78.7 (37/47)	79.9 (155/194)	12.9 (4/31)	97.3 (144/148)
**57.5-62.4**	2.8 (19/684)	0.5 (3/568)	17.6 (16/91)	34.9 (68/195)	3.0 (3/101)	71.4 (65/91)	72.3 (242/333)	8.1 (6/74)	95.0 (226/238)
**62.5-67.4**	5.4 (69/1288)	2.2 (21/961)	16.2 (47/290)	38.6 (124/321)	6.3 (9/142)	67.3 (113/168)	64.1 (221/345)	11.0 (10/91)	89.7 (195/217)
**67.5-72.4**	8.2 (134/1636)	2.0 (21/1057)	20.7 (111/535)	38.7 (176/455)	8.1 (15/185)	61.3 (157/256)	62.4 (284/455)	9.0 (9/100)	83.2 (252/303)
**72.5-77.4**	9.6 (141/1466)	2.6 (22/858)	20.2 (119/590)	39.6 (213/538)	8.9 (17/191)	56.4 (189/335)	59.7 (328/549)	8.9 (11/123)	78.3 (303/387)
**77.5-82.4**	13.2 (116/882)	5.0 (22/444)	21.6 (89/412)	35.0 (111/317)	5.7 (6/106)	50.5 (105/208)	61.7 (232/376)	15.5 (9/58)	73.3 (203/277)
**82.5-87.4**	14.2 (49/346)	5.9 (10/169)	22.0 (38/173)	30.1 (44/146)	4.3 (2/47)	43.6 (41/94)	55.5 (76/137)	8.7 (2/23)	68.0 (68/100)
**87.5-92.4**	12.5 (24/192)	2.7 (2/73)	21.7 (20/92)	28.9 (15/52)	18.2 (2/11)	31.7 (13/41)	53.3 (16/30)	0.0 (0/8)	76.2 (16/21)

The table shows the observed Tau-PET-positivity in the temporal cortex as a function of age and Aβ-status and stratified by syndrome diagnosis. This allows comparison with the prevalence of tau-positivity as estimated using logistic generalized estimating equation models presented in Table 2 of the main manuscript. The analyses presented in this table are based on 7,394 CU participants (68.7±11.1 years, 55.9% female) of which 7,186 had Aβ-status available (68.7±11.1 years, 56.0% female), 2,177 participants with MCI (71.3±8.8 years, 45.0% female) of which 2,121 had Aβ-status available (71.4±8.8 years, 44.8% female), and 2,477 participants with dementia (69.9±9.0 years, 50.9% female) of which 2,252 had Aβ-status available (69.9±9.0 years, 51.2% female).

Supplementary Table 4. Age by *APOE* ε4-dosage in cognitively unimpaired individuals

Age, y	% (95% CI)		
	***APOE* ε4 non-carrier**	***APOE* ε4 heterozygous**	***APOE* ε4 homozygous**
**50**	1.1<br>(0.7-1.5)	2.8<br>(1.7-3.8)	8.0<br>(5.1-11.0)
**55**	1.6<br>(1.0-2.1)	3.9<br>(2.6-5.3)	11.2<br>(7.6-14.7)
**60**	2.2<br>(1.6-2.9)	5.6<br>(3.9-7.2)	15.3<br>(11.0-19.6)
**65**	3.2<br>(2.4-4.0)	7.8<br>(5.7-9.9)	20.6<br>(15.6-25.7)
**70**	4.5<br>(3.6-5.5)	10.9<br>(8.3-13.4)	27.2<br>(21.3-33.1)
**75**	6.4<br>(5.2-7.7)	14.9<br>(11.7-18.2)	35.0<br>(28.3-41.7)
**80**	9.0<br>(7.4-10.6)	20.1<br>(16.0-24.3)	43.7<br>(36.3-51.0)
**85**	12.4<br>(10.2-14.7)	26.6<br>(21.4-31.9)	52.7<br>(44.8-60.6)
**90**	17.0<br>(13.8-20.2)	34.3<br>(27.8-40.8)	-

The prevalence estimates of tau-positivity in the temporal cortex were generated using logistic generalized estimating equation models including age and *APOE* ε4-dosage (*n*=6,288). Note that no prevalence estimates were provided if the indicated column included no participants. *APOE* = Apolipoprotein E; CI = Confidence interval; - = no participants in that age range.

Supplementary Table 5. Age by *APOE* ε4 genotype in cognitively unimpaired individuals

	% (95% CI)				
**Age, y**	**ε2ε3**	**ε2ε4**	**ε3ε3**	**ε3ε4**	**ε4ε4**
**50**	1.1 (0.6-1.6)	1.9 (0.7-3.0)	1.2 (0.7-1.6)	2.9 (1.8-4.1)	8.2 (5.2-11.2)
**55**	1.6 (0.9-2.2)	2.6 (1.1-4.2)	1.7 (1.1-2.2)	4.1 (2.7-5.6)	11.3 (7.7-15.0)
**60**	2.2 (1.3-3.1)	3.7 (1.7-5.8)	2.4 (1.7-3.1)	5.8 (4.1-7.6)	15.5 (11.2-19.8)
**65**	3.1 (2.0-4.3)	5.3 (2.6-8.0)	3.4 (2.5-4.2)	8.2 (6.0-10.4)	20.8 (15.7-25.9)
**70**	4.4 (3.0-5.9)	7.4 (3.9-11.0)	4.8 (3.7-5.8)	11.3 (8.5-14.1)	27.4 (21.5-33.2)
**75**	6.2 (4.3-8.2)	10.3 (5.7-14.8)	6.7 (5.4-8.0)	15.4 (12.0-18.9)	35.1 (28.4-41.7)
**80**	8.7 (6.1-11.3)	14.1 (8.3-19.9)	9.3 (7.6-11.0)	20.7 (16.3-25.2)	43.7 (36.3-51.0)
**85**	12.0 (8.6-15.5)	19.1 (11.9-26.2)	12.8 (10.4-15.2)	27.3 (21.6-32.9)	52.6 (44.7-60.5)
**90**	16.4 (11.8-21.1)	25.3 (16.6-33.9)	17.4 (14.0-20.8)	35.0 (28.0-42.0)	-

The prevalence estimates of tau-positivity in the temporal cortex were generated using logistic generalized estimating equation models including age and *APOE* ε4 genotype (*n*=5,963). Note that no prevalence estimates were provided if the indicated column included no participants and that there were no *APOE* ε2ε2 tau-positive cognitively unimpaired individuals in our sample. *APOE* = Apolipoprotein E; CI = Confidence interval; - = no participants in that age range.

Supplementary Table 6. Tau-PET-positivity in association with age, Aβ- and *APOE* ε4-status

	CU	MCI	Dementia
**Age**	β=0.08<br>p<0.001	β=0.05<br>p<0.001	β=0.03<br>p=0.11
**Aβ-status**	β=2.24<br>p<0.001	β=2.77<br>p<0.001	β=3.54<br>p<0.001
***APOE* ε4-status**	β=0.55<br>p<0.001	β=0.64<br>p<0.001	β=0.59<br>p<0.001
**Age * Aβ-status**	β=-0.05<br>p<0.001	β=-0.08<br>p<0.001	β=-0.10<br>p<0.001

Model outputs for logistic generalized estimating equation models including age, Aβ-status, *APOE* ε4-status and an interaction term between age and Aβ-status as predictors of tau-positivity in the temporal cortex. Models were performed stratified for CU *(n*=6,384)*,* MCI (*n*=1,823) and dementia *(n*=1,869)*.* Aβ = Amyloid-beta; *APOE* = Apolipoprotein E; CU = Cognitively unimpaired; MCI = mild cognitive impairment.

Supplementary Table 7. Tau-PET-positivity in association with age, Aβ-status and sex

	CU	MCI	Dementia
**Age**	β=0.07<br>p<0.001	β=0.05<br>p<0.001	β=0.03<br>p=0.11
**Aβ-status**	β= 2.48<br>p<0.001	β=2.96<br>p<0.001	β=3.68<br>p<0.001
**Sex (f)**	β=-0.005<br>p=0.97	β=0.34<br>p<0.001	β=0.59<br>p=0.004
**Age * Aβ-status**	β=-0.06<br>p<0.001	β=-0.08<br>p<0.001	β=-0.09<br>p<0.001
**Sex (f) * Aβ-status**	β=0.34<br>p=0.02	n.s.	n.s.

Model outputs for logistic generalized estimating equation models including age, Aβ-status, sex and interaction terms between age and Aβ-status, and between sex and Aβ-status as predictors of tau-positivity in the temporal cortex (CU and MCI), and age, Aβ-status, sex and an interaction term between age and Aβ-status (dementia). Models were performed stratified for CU (*n*=7,185), MCI (*n*=2,121) and dementia (*n*=2,252). Aβ = Amyloid-beta; CU = Cognitively unimpaired; MCI = Mild cognitive impairment; n.s. = Not significant.

Supplementary Table 8. Prevalence estimates of tau-positivity in independent post-mortem data

	CU, % (95% CI)		MCI, % (95% CI)		Dementia, % (95% CI)	
**Age, y**	**Aβ-negative**	**Aβ-positive**	**Aβ-negative**	**Aβ-positive**	**Aβ-negative**	**Aβ-positive**
**60**	0.0<br>(0.0-0.0)	2.6<br>(0.0-7.9)	0.4<br>(0.1-0.6)	45.6<br>(0.0-98.0)	2.4<br>(0.0-9.5)	78.9<br>(71.2-86.5)
**65**	0.0<br>(0.0-0.0)	3.6<br>(0.0-9.9)	0.6<br>(0.3-0.8)	43.9<br>(0.0-89.2)	2.7<br>(0.0-9.4)	76.7<br>(68.9-84.5)
**70**	0.0<br>(0.0-0.0)	4.9<br>(0.0-12.0)	0.8<br>(0.5-1.0)	42.8<br>(2.7-82.8)	3.0<br>(0.0-9.2)	74.4<br>(66.4-82.4)
**75**	0.0<br>(0.0-0.1)	6.5<br>(0.0-14.4)	1.2<br>(0.9-1.4)	41.0<br>(8.6-73.4)	3.3<br>(0.0-8.9)	72.0<br>(63.8-80.2)
**80**	0.1<br>(0.0-0.3)	8.6<br>(0.2-16.9)	1.7<br>(1.3-2.1)	39.6<br>(13.4-65.8)	3.7<br>(0.0-8.5)	69.4<br>(61.1-77.8)
**85**	0.3<br>(0.0-0.9)	11.3<br>(2.9-19.7)	2.4<br>(1.5-3.3)	38.2<br>(17.8-58.5)	4.1<br>(0.0-8.2)	66.7<br>(58.1-75.4)
**90**	0.7<br>(0.0-2.3)	14.7<br>(6.9-22.6)	3.5<br>(1.5-5.4)	36.8<br>(21.7-51.8)	4.5<br>(0.6-8.4)	63.9<br>(55.0-72.9)
**95**	1.8<br>(0.0-6.2)	19.0<br>(11.9-26.0)	4.9<br>(1.3-8.5)	35.4<br>(24.5-46.3)	5.0<br>(0.4-9.6)	61.0<br>(51.7-70.3)
**100**	4.8<br>(0.0-16.0)	24.1<br>(17.0-31.2)	7.0<br>(0.8-13.2)	34.0<br>(24.9-43.2)	5.5<br>(0.0-11.8)	58.0<br>(48.3-67.8)

The prevalence estimates of tau-positivity defined as Braak stage V-VI neurofibrillary tangle pathology were generated using logistic generalized estimating equation models including age, Aβ-status and an interaction term between age and Aβ-status, and models were stratified by syndrome diagnosis. Note that there were only a few individuals with an age-at-death <60 years (i.e., CU n=15, MCI n=8, and dementia n=116 dementia) hence the lowest age estimate in this Table starts at age 60. Prevalence estimates are based on 1,026 CU, 661 MCI and 3,385 dementia participants. Aβ = Amyloid-beta; CU = Cognitively unimpaired; CI = Confidence interval; MCI = Mild cognitive impairment

Supplementary Figure 5. Prevalence of Tau-positivity on PET (whole-brain ROI) vs neuropathological examination (Braak V-VI)

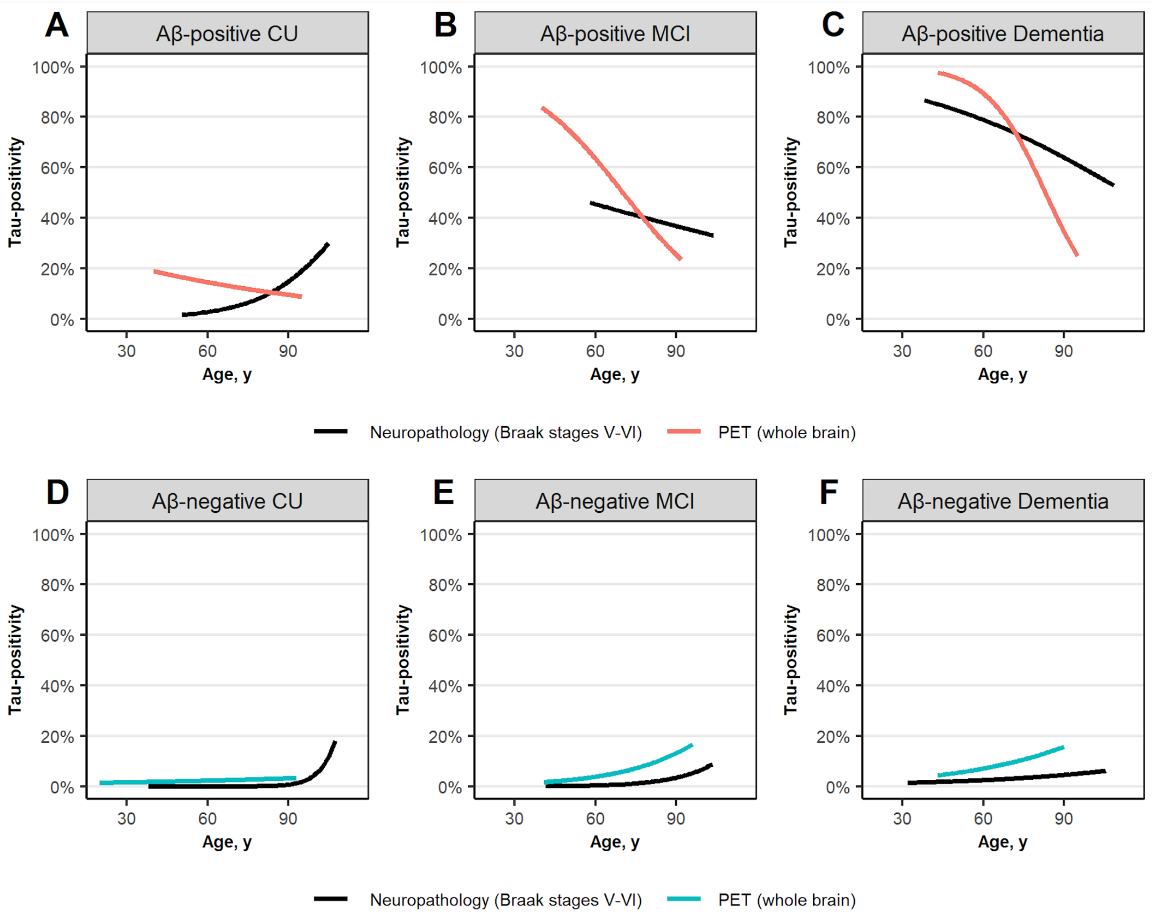

Tau-positivity on PET or neuropathology in CU (panels A and D), MCI (panels B and E) and dementia (panels C and F) was modelled using age, Aβ-status and an interaction between age and Aβ-status. Models were stratified by syndrome diagnosis. The y-axes reflect estimated probabilities of Tau-positivity on PET (whole brain) or neuropathology (Braak V-VI) (prevalence estimates) generated from generalized estimating equations. Prevalence estimates in Aβ-positive participants are shown in panels A-C, and prevalence estimates in Aβ-negative participants are shown in panels D-F. Prevalence estimates for PET are based on 7,186 CU, 2,121 MCI and 2,252 dementia participants. Prevalence estimates for neuropathology are based on 1,026 CU, 661 MCI and 3,385 dementia participants. Aβ = Amyloid-beta; CU = Cognitively unimpaired; MCI, mild cognitive impairment; PET = Positron emission tomography.

Supplementary Table 9. Cohort-specific Aβ-status information

	n	n missing Aβ-status (%)	Aβ+ (% of known status)	Modality	Measure	Method	Threshold
**A4 study**	447	0 (0.0)	380 (85.0)	PET	[^{18}F]FBP	Centiloid	20
**ADNI**	866	18 (2.1)	359 (42.3)	PET	[^{18}F]FBP, [^{18}F]FBB	Centiloid	20
**ADNI-DOD**	98	0 (0.0)	38 (38.8)	PET	[^{18}F]FBP	SUVR	0.79
**Amsterdam-ADC**	186	3 (1.6)	126 (68.9)	PET (61%)	[^{18}F]FBP, [^{18}F]FMM, [^{18}F]FBB, [^{11}C]PiB	Visual read	-
				CSF	Aβ42	ELISA	813
**Amsterdam-Twin Study**	80	0 (0.0)	31 (38.8)	PET	[^{18}F]FMM	Visual reads	-
**Austin Health-FTP**	80	0 (0.0)	36 (45.0)	PET	[^{18}F]NAV4694	Centiloid	20
**Austin Health-MK**	755	1 (0.1)	410 (54.4)	PET	[^{18}F]NAV4694	Centiloid	25
**Avid cohorts**	510	1 (0.2)	323 (63.5)	PET	[^{18}F]FBP	Centiloid	20
**BACS**	134	0 (0.0)	56 (41.8)	PET	[^{11}C]PiB	DVR	1.065
**Barcelona-Beta**	99	0 (0.0)	48 (48.5)	PET	[^{18}F]FMM	Centiloid	12
				CSF	Aβ42/40 / p-tau181	Elecsys	<0.071 / >24
**BioFINDER-1**	242	40 (16.5)	131 (64.9)	PET (93%)	[^{18}F]FMM	Centiloid	20
				CSF	Aβ42	ELISA	510
**BioFINDER-2**	1817	21 (1.2)	768 (42.8)	PET (57%)	[^{18}F]FMM	Centiloid	20
				CSF	Aβ42	ELISA	510
**Cambridge**	83	39 (47.0)	25 (56.8)	PET (95%)		Centiloid	19
				CSF	Aβ42	ELISA	
**Cologne**	38	2 (5.3)	30 (83.3)	PET (52%)	[^{11}C]PiB	DVR	1.2
				CSF			
**Columbia University**	462	0 (0.0)	43 (9.3)	PET	[^{18}F]FBB	Visual read	-
**CPAS**	476	18 (3.8)	216 (47.2)	PET	[^{18}F]FBP	Visual read	-
**Gothenburg**	68	44 (64.7)	15 (62.5)	CSF	Aβ42	ELISA	981
**HABS**	194	0 (0.0)	64 (33.0)	PET	[^{11}C]PiB	DVR	1.2
**Indiana University**	147	12 (8.2)	52 (38.5)	PET		Centiloid	21.02
**LEADS**	420	4 (1.0)	276 (66.3)	PET	[^{18}F]FBB	Centiloid	20
**Leipzig**	42	25 (59.5)	17 (100.0)	PET	[^{18}F]FBB, [^{18}F]FMM	Visual reads	-

**Leuven**	36	2 (5.6)	3 (8.8)	PET	[^{11}C]PiB	SUVR	1.3
**Louvain**	174	2 (1.1)	95 (55.2)	PET (51%)	[^{18}F]FMM	Centiloid	25
				CSF			
					Aβ42	LUMIPULSE	437
**MCSA**	1614	0 (0.0)	581 (36.0)	PET	[^{11}C]PiB	SUVR	1.48
**Munich**	280	92 (32.9)	69 (36.7)	PET (60%)	[^{18}F]FBB, [^{18}F]FMM	Visual read	-
				CSF	Aβ42/40 or Aβ42	ELISA	5.5% / 375
**OASIS**	420	0 (0.0)	151 (36.0)	PET	[^{11}C]PiB	Centiloid	20
**Paris**	104	1 (1.0)	58 (56.3)	PET	[^{11}C]PiB	SUVR	1.4
**Prevent-AD**	250	0 (0.0)	84 (33.6)	PET	[^{18}F]NAV4694	SUVR	1.26
**Seoul**	104	0 (0.0)	77 (74.0)	PET	[^{11}C]PiB	Centiloid	25
**Stanford**	94	0 (0.0)	53 (56.4)	PET (57%)	[^{18}F]FBB	Centiloid	18
				CSF	Aβ42/Aβ40	LUMIPULSE	0.0752
**TRIAD**	420	0 (0.0)	169 (40.2)	PET	[^{18}F]NAV4694	SUVR	1.55
**UCL**	151	0 (0.0)	68 (45.0)	PET	[^{18}F]FBP	Centiloid	11.9
**UCSF**	197	31 (15.7)	97 (58.4)	PET	[^{11}C]PiB	Centiloid	20
**UHG**	278	21 (7.6)	141 (54.9)	PET (85%)	[^{18}F]FBP, [^{18}F]FMM	Visual read	-
				CSF	Aβ42	ELISA	880.5
**WRAP**	271	1 (0.4)	48 (17.8)	PET	[^{11}C]PiB	Centiloid	20

Aβ=Amyloid-beta; CSF = Cerebrospinal fluid; DVR=distribution volume ratio; FBB = Florbetaben; FBP = Florbetapir; FMM = Flutemetamol; PIB = Pittsburg compound-B; SUVR=standardized uptake value ratio, DVR=distribution volume ratio; ADNI = Alzheimer’s disease neuroimaging initiative; ADNI-DOD = Alzheimer’s disease neuroimaging initiative department of defense; ADC = Amsterdam dementia cohort; BACS = Berkeley aging cohort study; CPAS = Chinese Preclinical Alzheimer's disease Study; HABS = Harvard aging brain study; LEADS = Longitudinal early-onset Alzheimer's disease study; MCSA = Mayo clinic study of aging; OASIS = Open access series of imaging studies; TRIAD = Translational biomarkers in aging and dementia; UCL = University college London; UCSF = University of California San Francisco; UHG = University hospitals Geneva; WRAP = Wisconsin registry for Alzheimer's prevention.

Supplementary Table 10. Cohort-specific Tau-PET information

Cohort	Tracer	Scan interval, min	Metric	Reference region
**A4 study**	[^{18}F]flortaucipir	80-110	SUVR	Inf. Cerebellar cortex
**ADNI**	[^{18}F]flortaucipir	75–105	SUVR	Inf. Cerebellar cortex
**ADNI-DOD**	[^{18}F]flortaucipir	75–105	SUVR	Inf. Cerebellar cortex
**Amsterdam-ADC**	[^{18}F]flortaucipir	80-100	SUVR	Cerebellar cortex
**Amsterdam-Twin study**	[^{18}F]flortaucipir	80-100	SUVR	Cerebellar cortex
**Austin Health-FTP**	[^{18}F]flortaucipir	80-100	SUVR	Cerebellar cortex
**Austin Health-MK**	[^{18}F]MK6240	90-110	SUVR	Cerebellar cortex
**Avid cohorts**	[^{18}F]flortaucipir	75-105	SUVR	Subject-specific white matter (PERSI)
**BACS**	[^{18}F]flortaucipir	80-100	SUVR	Inf. Cerebellar cortex
**Barcelona-Beta**	[^{18}F]RO948	70-90	SUVR	Inf. Cerebellar cortex
**BioFINDER-1**	[^{18}F]flortaucipir	80-100	SUVR	Inf. Cerebellar cortex
**BioFINDER-2**	[^{18}F]RO948	70-90	SUVR	Inf. Cerebellar cortex
**Cambridge**	[^{18}F]flortaucipir	0-90	BP_{ND}	Sup. Cerebellar cortex
**Cologne-PI**	[^{18}F]PI2620	0-90	BP_{ND}	Paracentral gyrus
**Cologne-FTP**	[^{18}F]flortaucipir	90-105	SUVR	Cerebellar cortex
**Columbia University**	[^{18}F]MK6240	90-110	SUVR	Inf. Cerebellar cortex
**CPAS**	[^{18}F]MK6240	90-110	SUVR	Inf. Cerebellar cortex
**Gothenburg**	[^{18}F]RO948	70-90	SUVR	Inf. Cerebellar cortex
**HABS**	[^{18}F]flortaucipir	80-100	SUVR	Cerebellar cortex
**Indiana University**	[^{18}F]flortaucipir	75–105	SUVR	Cerebellar crus
**LEADS**	[^{18}F]flortaucipir	75–105	SUVR	Inf. Cerebellar cortex
**Leipzig**	[^{18}F]PI2620	0–60	DVR	Cerebellar cortex
**Leuven**	[^{18}F]MK6240	90-120	SUVR	Cerebellar cortex
**Louvain**	[^{18}F]MK6240	90-120	SUVR	Cerebellar cortex
**MCSA**	[^{18}F]flortaucipir	80-100	SUVR	Cerebellar crus

**MK cohort**	[^{18}F]MK6240	90-110	SUVR	Inf. Cerebellar cortex
**Munich**	[^{18}F]PI2620	0–60	DVR	Inf. Cerebellar cortex
**OASIS**	[^{18}F]flortaucipir	80-100	SUVR	Cerebellar cortex
**Paris**	[^{18}F]flortaucipir	80-100	SUVR	Cerebellar cortex
**Prevent-AD**	[^{18}F]flortaucipir	80-100	SUVR	Inf. Cerebellar cortex
**Seoul**	[^{18}F]flortaucipir	80-100	SUVR	Cerebellar cortex
**Stanford**	[^{18}F]PI2620	45-75 or 60-90*	SUVR	Inf. Cerebellar cortex
**TRIAD**	[^{18}F]MK6240	90-110	SUVR	Inf. Cerebellar cortex
**UCL**	[^{18}F]MK6240	90-110	SUVR	Inf. Cerebellar cortex
**UCSF**	[^{18}F]flortaucipir	80-100	SUVR	Inf. Cerebellar cortex
**UHG**	[^{18}F]flortaucipir	80-100	SUVR	Inf. Cerebellar cortex
**WRAP**	[^{18}F]MK6240	70-90	SUVR	Inf. Cerebellar cortex

BP_{ND}=binding potential (non-displacable); DVR=distribution volume ratio; SUVR=standardized uptake value ratio; Inf = Inferior; ADNI = Alzheimer’s disease neuroimaging initiative; ADNI-DOD = Alzheimer’s disease neuroimaging initiative department of defense; ADC = Amsterdam dementia cohort; BACS = Berkeley aging cohort study; CPAS = Chinese Preclinical Alzheimer's disease Study; HABS = Harvard aging brain study; LEADS = Longitudinal early-onset Alzheimer's disease study; MCSA = Mayo clinic study of aging; OASIS = Open access series of imaging studies; TRIAD = Translational biomarkers in aging and dementia; UCL = University college London; UCSF = University of California San Francisco; UHG = University hospitals Geneva; WRAP = Wisconsin registry for Alzheimer's prevention.
*60-90 [^{18}F]PI2620 data were interpolated to a 45-75 min scale using methods described in Pontecorvo et al. 2019 (PMID: 31009046)

Supplementary Table 11. Cohort-specific Tau-PET region-of-interest compositions

Cohort	Atlas	Temporal Meta-ROI	Entorhinal cortex	Whole brain	Volume weighted
**A4 study**	Desikan Killiany	Standard*	Entorhinal cortex	All	Yes
**ADNI**	Desikan Killiany	Standard*	Entorhinal cortex	All	Yes
**ADNI-DOD**	Desikan Killiany	Standard*	Entorhinal cortex	All	Yes
**Amsterdam-ADC**	Desikan Killiany	Standard*	Entorhinal cortex	All	Yes
**Amsterdam-Twin study**	Hammers and Svarer atlas	Entorhinal, fusiform, inferior and middle temporal gyrus, parahippocampal gyrus, amygdala	Entorhinal cortex	All	Yes
**Austin Health-FTP**	Desikan Killiany	Standard*	Entorhinal cortex	All	Yes
**Austin Health-MK**	Desikan Killiany	Standard*	Entorhinal cortex	All	Yes
**Avid cohorts**	Desikan Killiany	Standard*	Entorhinal cortex	All	Yes
**BACS**	Desikan Killiany	Standard*	Entorhinal cortex	All	Yes
**Barcelona-Beta**	AAL	Standard*	Entorhinal cortex	All	Yes
**BioFINDER-1**	Desikan Killiany	Standard*	Entorhinal cortex	All	Yes
**BioFINDER-2**	Desikan Killiany	Standard*	Entorhinal cortex	All	Yes
**Cambridge**	Schaefer 200	49 areas, see #	Areas 57, 164 (Entorhinal cortex)	All	No
**Cologne**	Desikan Killiany	Standard*	Entorhinal cortex	All	Yes
**Columbia University**	Desikan Killiany	Standard*	Entorhinal cortex	All	Yes
**CPAS**	Desikan Killiany	Braak I-IV	Entorhinal cortex	Braak I-VI	No
**Gothenburg**	Desikan Killiany	Entorhinal, fusiform, inferior temporal, middle temporal, parahippocampal	Entorhinal cortex	All except amygdala	No
**HABS**	Desikan Killiany	Standard*	Entorhinal cortex	All	Yes
**Indiana University**	Desikan Killiany	Standard*	Entorhinal cortex	All	Yes
**LEADS**	Desikan Killiany	Standard*	Entorhinal cortex	All	Yes
**Leipzig**	Desikan Killiany	Standard*	Entorhinal cortex	All	No
**Leuven**	Brainnetome atlas	Area 28 34, Entorhinal cortex, Medial amygdala, Lateral amygdala, Rostroventral area 20, Medioventral area 37, Lateroventral area 37, Caudal area 21, Rostral	Areas 28 34 (Entorhinal cortex)	All	No

		area 21, Dorsolateral area 37, Anterior Superior Temporal Sulcus, Intermediate ventral area 20, Extreme lateroventral area 37, Rostral area 20, Intermediate lateral area 20, Ventrolateral area 37, Caudolateral area 20, Caudoventral area 20			
**Louvain**	Desikan Killiany	Standard*	Entorhinal cortex	All	Yes
**MCSA**	Mayo MCALT atlas	Prefrontal, orbitofrontal, parietal, temporal, anterior and posterior cingulate, and precuneus	Entorhinal cortex	All	Yes
**MK cohort**	Desikan Killiany	Standard*	Entorhinal cortex	All	Yes
**Munich**	Desikan Killiany	Standard*	Entorhinal cortex	All	No
**OASIS**	Desikan Killiany	Standard*	Entorhinal cortex	All	Yes
**Paris**	Desikan Killiany	Standard*	Entorhinal cortex	All	Yes
**Prevent-AD**	Desikan Killiany & AAL	Standard*	Entorhinal cortex	All	Yes
**Seoul**	Desikan Killiany	Standard*	Entorhinal cortex	All	No
**Stanford**	Desikan Killiany	Entorhinal, amygdala, inferior temporal	Entorhinal cortex	Composite^	Yes
**TRIAD**	Desikan Killiany	Standard*	Entorhinal cortex	All	
**UCL**	Desikan Killiany	Standard*	Entorhinal cortex	All	Yes
**UCSF**	Desikan Killiany	Standard*	Entorhinal cortex	All	Yes
**UHG**	Desikan Killiany	Standard*	Entorhinal cortex	All	Yes
**WRAP**	Desikan Killiany	Standard*	Entorhinal cortex	All	Yes

ERC = entorhinal cortex; ROI = Region-of-interest; MCSALT = Mayo Clinic Adult Lifespan Template and Atlases; ADNI = Alzheimer's disease neuroimaging initiative; ADNI-DOD = Alzheimer's disease neuroimaging initiative Department of Defense; ADC = Amsterdam dementia cohort; BACS = Berkeley aging cohort study; CPAS = Chinese Preclinical Alzheimer's disease Study; HABS = Harvard aging brain study; LEADS = Longitudinal early-onset Alzheimer's disease study; MCSA = Mayo clinic study of aging; OASIS = Open access series of imaging studies; TRIAD = Translational biomarkers in aging and dementia; UCL = University college London; UCSF = University of California San Francisco; UHG = University hospitals Geneva; WRAP = Wisconsin registry for Alzheimer's prevention. *=Standard Desikan Killiany ROIs for temporal meta-ROI: bilateral entorhinal, amygdala, fusiform, parahippocampus and inferior and middle temporal cortex. #=Schaefer regions: 1, 2, 3, 4, 6, 17, 31, 32, 47, 49, 52, 53, 57, 58, 64, 72, 73, 74, 75, 77, 84, 88, 90, 98, 100, 101, 102, 103, 104, 106, 107, 110, 118, 123, 135, 136, 148, 155, 163, 164, 168, 169, 178, 179, 180, 186, 188, 192, 193. ^=Entorhinal, amygdala, inferior temporal, inferior parietal, precuneus, rostral middle frontal, precentral.

Supplementary Table 12. Cohort- and tracer-specific Tau-PET thresholds

Cohort	Tau PET tracer	Cohort specific threshold TMR	Tracer specific GMM TMR	Cohort specific threshold ERC	Tracer specific GMM ERC	Cohort specific threshold WB	Tracer specific GMM WB
**A4 study**	[^{18}F]flortaucipir	1.26	1.40	1.27	1.30	1.17	1.34
**ADNI**	[^{18}F]flortaucipir	1.35	1.40	1.34	1.30	1.22	1.34
**ADNI-DOD**	[^{18}F]flortaucipir	1.25	1.40	1.19	1.30	1.14	1.34
**Amsterdam-ADC**	[^{18}F]flortaucipir	1.30	1.40	1.31	1.30	1.20	1.34
**Amsterdam-Twin study**	[^{18}F]flortaucipir	1.28	1.40	1.23	1.30	1.19	1.34
**Austin Health-FTP**	[^{18}F]flortaucipir	1.32	1.40	1.36	1.30	1.21	1.34
**Austin Health-MK**	[^{18}F]MK6240	1.22	1.43	1.53	1.38	1.10	1.31
**Avid cohorts**	[^{18}F]flortaucipir	1.30	1.40	1.33	1.30	1.23	1.34
**BACS**	[^{18}F]flortaucipir	1.31	1.40	1.31	1.30	1.23	1.34
**Barcelona-Beta**	[^{18}F]RO948	1.34	1.41	1.32	1.39	1.28	1.28
**BioFINDER-1**	[^{18}F]flortaucipir	1.26	1.40	1.23	1.30	1.17	1.34
**BioFINDER-2**	[^{18}F]RO948	1.35	1.41	1.46	1.39	1.21	1.28
**Cambridge**	[^{18}F]flortaucipir	1.32	1.40	1.29	1.30	1.24	1.34
**Cologne-FTP**	[^{18}F]flortaucipir	1.32	1.40	1.29	1.30	1.24	1.34
**Cologne-PI**	[^{18}F]PI2620	1.36	1.41	1.59	1.34	1.28	1.31
**Columbia University**	[^{18}F]MK6240	1.55	1.43	1.90	1.38	1.42	1.31
**Gothenburg**	[^{18}F]RO948	1.36	1.41	1.43	1.39	1.24	1.28
**CPAS**	[^{18}F]MK6240	1.08	1.43	1.05	1.38	1.07	1.31
**HABS**	[^{18}F]flortaucipir	1.29	1.40	1.28	1.30	1.22	1.34
**Indiana University**	[^{18}F]flortaucipir	1.27	1.40	1.28	1.30	1.27	1.34
**LEADS**	[^{18}F]flortaucipir	1.25	1.40	1.26	1.30	1.20	1.34
**Leipzig**	[^{18}F]PI2620	1.36	1.41	1.59	1.34	1.28	1.31
**Leuven**	[^{18}F]MK6240	1.42	1.43	1.32	1.38	1.36	1.31
**Louvain**	[^{18}F]MK6240	1.38	1.43	1.94	1.38	1.21	1.31
**MCSA**	[^{18}F]flortaucipir	1.35	1.40	1.28	1.30	1.29	1.34

**MK cohorts**	[^{18}F]MK6240	1.25	1.43	1.34	1.38	1.17	1.31
**Munich**	[^{18}F]PI2620	1.29	1.41	1.41	1.34	1.22	1.31
**OASIS**	[^{18}F]flortaucipir	1.29	1.40	1.27	1.30	1.20	1.34
**Paris**	[^{18}F]flortaucipir	1.46	1.40	1.38	1.30	1.46	1.34
**Prevent-AD**	[^{18}F]flortaucipir	1.29	1.40	1.25	1.30	1.18	1.34
**Seoul**	[^{18}F]flortaucipir	1.44	1.40	1.53	1.30	1.27	1.34
**Stanford**	[^{18}F]PI2620	1.40	1.41	1.69	1.34	1.32	1.31
**TRIAD**	[^{18}F]MK6240	1.13	1.43	1.23	1.38	1.16	1.31
**UCL**	[^{18}F]MK6240	1.41	1.43	1.47	1.38	1.14	1.31
**UCSF**	[^{18}F]flortaucipir	1.31	1.40	1.31	1.30	1.23	1.34
**UHG**	[^{18}F]flortaucipir	1.35	1.40	1.35	1.30	1.28	1.34
**WRAP**	[^{18}F]MK6240	1.26	1.43	1.41	1.38	1.16	1.31

Cohort specific thresholds were based on the mean+2SDs in amyloid-β-negative cognitively unimpaired individuals aged >50 years from the same cohort. For three cohorts that did not include a sufficient number amyloid-β-negative cognitively unimpaired individuals aged >50 years (Cologne, Cambridge and Leipzig), we used a tracer-specific average across cohorts weighted by the sample size of each cohort. GMM = Gaussian mixture modelling, performed across all cohorts using the same tracer. TMR = Temporal meta ROI, WB = Whole brain; ERC = Entorhinal cortex; ADNI = Alzheimer's disease neuroimaging initiative; ADNI-DOD = Alzheimer's disease neuroimaging initiative department of defense; ADC = Amsterdam dementia cohort; BACS = Berkeley aging cohort study; CPAS = Chinese Preclinical Alzheimer's disease Study; HABS = Harvard aging brain study; LEADS = Longitudinal early-onset Alzheimer's disease study; MCSA = Mayo clinic study of aging; OASIS = Open access series of imaging studies; TRIAD = Translational biomarkers in aging and dementia; UCL = University college London; UCSF = University of California San Francisco; UHG = University hospitals Geneva; WRAP = Wisconsin registry for Alzheimer's prevention.

Supplementary Figure 6. Cohort-specific histograms of Tau PET SUVRs in the temporal cortex in Aβ- CU individuals

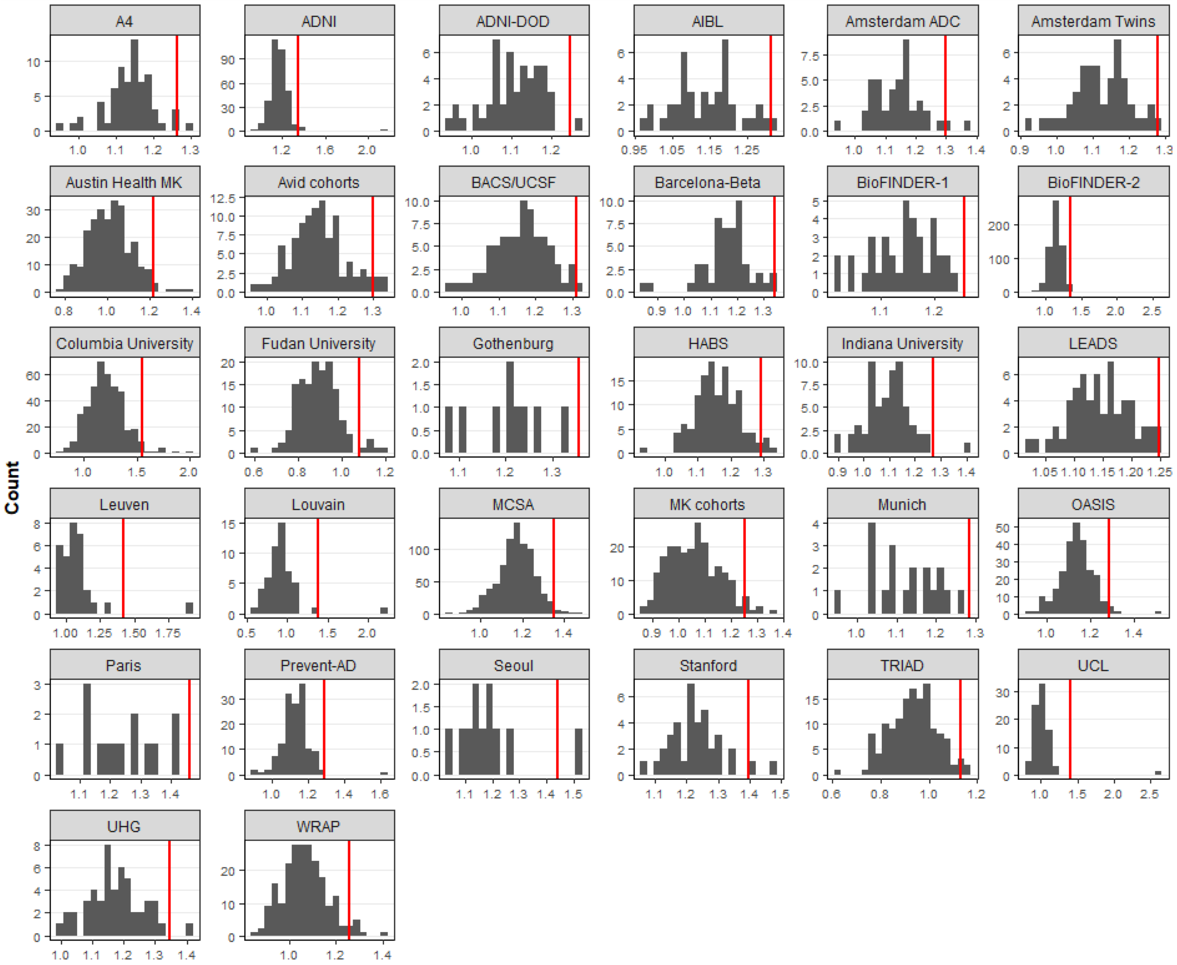

Distribution of Tau-PET SUVR's (temporal cortex) in Aβ- cognitively unimpaired individuals older than 50 years that served as the reference group to determine the Tau-PET positivity thresholds used in the primary analyses in the manuscript.
