# Supplementary material for: Tau PET positivity in individuals with and without cognitive impairment varies with age, amyloid-β status, APOE genotype and sex
Source: Nat Neurosci. 2025 Jul 16;28(8):1610–21. doi: 10.1038/s41593-025-02000-6 (PMC12321570; doi:10.1038/s41593-025-02000-6)
Supplement: Supplementary file 2 — Reporting Summary [file 41593_2025_2000_MOESM2_ESM.pdf]

nature portfolio

Corresponding author(s): Rik Ossenkoppele

Last updated by author(s): Apr 7, 2025

Reporting Summary

Nature Portfolio wishes to improve the reproducibility of the work that we publish. This form provides structure for consistency and transparency in reporting. For further information on Nature Portfolio policies, see our Editorial Policies and the Editorial Policy Checklist.

Statistics

For all statistical analyses, confirm that the following items are present in the figure legend, table legend, main text, or Methods section.

n/a	Confirmed	
☐	☒	The exact sample size (*n*) for each experimental group/condition, given as a discrete number and unit of measurement
☐	☒	A statement on whether measurements were taken from distinct samples or whether the same sample was measured repeatedly
☐	☒	The statistical test(s) used AND whether they are one- or two-sided<br>*Only common tests should be described solely by name; describe more complex techniques in the Methods section.*
☐	☒	A description of all covariates tested
☐	☒	A description of any assumptions or corrections, such as tests of normality and adjustment for multiple comparisons
☐	☒	A full description of the statistical parameters including central tendency (e.g. means) or other basic estimates (e.g. regression coefficient) AND variation (e.g. standard deviation) or associated estimates of uncertainty (e.g. confidence intervals)
☐	☒	For null hypothesis testing, the test statistic (e.g. *F*, *t*, *r*) with confidence intervals, effect sizes, degrees of freedom and *P* value noted<br>*Give P values as exact values whenever suitable.*
☒	☐	For Bayesian analysis, information on the choice of priors and Markov chain Monte Carlo settings
☒	☐	For hierarchical and complex designs, identification of the appropriate level for tests and full reporting of outcomes
☒	☐	Estimates of effect sizes (e.g. Cohen's *d*, Pearson's *r*), indicating how they were calculated

Our web collection on statistics for biologists contains articles on many of the points above.

Software and code

Policy information about availability of computer code

Data collection	implemented in R version 4.2.1 and code can be requested from the corresponding authors
Data analysis	implemented in R version 4.2.1 and codes are freely available: https://github.com/OssenKoppeLab

For manuscripts utilizing custom algorithms or software that are central to the research but not yet described in published literature, software must be made available to editors and reviewers. We strongly encourage code deposition in a community repository (e.g. GitHub). See the Nature Portfolio guidelines for submitting code & software for further information.

Data

Policy information about availability of data

All manuscripts must include a data availability statement. This statement should provide the following information, where applicable:

- Accession codes, unique identifiers, or web links for publicly available datasets
- A description of any restrictions on data availability
- For clinical datasets or third party data, please ensure that the statement adheres to our policy

Due to the multicenter design of the study, individual participant data from each cohort will have to be made available through the PIs of the respective cohorts. Generally, anonymized data can be shared by request from qualified academic investigators for the purpose of replicating procedures and results presented in the article, if the data transfer is in agreement with the data protection regulation at the institution and is approved by the local Ethics Review Board. ata availability statement is included in the manuscript

Research involving human participants, their data, or biological material

Policy information about studies with human participants or human data. See also policy information about sex, gender (identity/presentation), and sexual orientation and race, ethnicity and racism.

Reporting on sex and gender	Self-reported sex and/or gender information across centers is included in table 1
Reporting on race, ethnicity, or other socially relevant groupings	A subset of centers reported self-reported sex and/or ethnicity status and this information is included in table 1
Population characteristics	Included in manuscript, table 1
Recruitment	Recruitment strategies varied by center, information is included in previous publication
Ethics oversight	Data collection oversight by local IRBs data anlaysis by Ethics Committee of Lund University

Note that full information on the approval of the study protocol must also be provided in the manuscript.

Field-specific reporting

Please select the one below that is the best fit for your research. If you are not sure, read the appropriate sections before making your selection.

☒ Life sciences ☐ Behavioural & social sciences ☐ Ecological, evolutionary & environmental sciences

For a reference copy of the document with all sections, see nature.com/documents/nr-reporting-summary-flat.pdf

Life sciences study design

All studies must disclose on these points even when the disclosure is negative.

Sample size	12048
Data exclusions	No data excluded from participants that met in- and exclusion criteria
Replication	No
Randomization	n.a.
Blinding	n.a.

Reporting for specific materials, systems and methods

We require information from authors about some types of materials, experimental systems and methods used in many studies. Here, indicate whether each material, system or method listed is relevant to your study. If you are not sure if a list item applies to your research, read the appropriate section before selecting a response.

Materials & experimental systems

n/a	Involved in the study	
☒	☐	Antibodies
☒	☐	Eukaryotic cell lines
☒	☐	Palaeontology and archaeology
☒	☐	Animals and other organisms
☒	☐	Clinical data
☒	☐	Dual use research of concern
☒	☐	Plants

Methods

n/a	Involved in the study	
☒	☐	ChIP-seq
☒	☐	Flow cytometry
☐	☒	MRI-based neuroimaging

Magnetic resonance imaging

Experimental design

Design type	Processing was performed with various software at participating center (included in supplement)

Design specifications | see above

Behavioral performance measures | na

Acquisition

Imaging type(s) | Structural

Field strength | Various

Sequence & imaging parameters | Various

Area of acquisition | Cranium

Diffusion MRI ☐ Used ☒ Not used

Preprocessing

Preprocessing software | Processing was performed with various software at participating center (included in supplement)

Normalization | See above

Normalization template | See above

Noise and artifact removal | See above

Volume censoring | See above

Statistical modeling & inference

Model type and settings | Logistic generalized estimating equation (GEE)

Effect(s) tested | Age, amyloid-status, sex, APOE genotype

Specify type of analysis: ☐ Whole brain ☐ ROI-based ☒ Both

Anatomical location(s) | *Describe how anatomical locations were determined (e.g. specify whether automated labeling algorithms or probabilistic atlases were used).*

Statistic type for inference | Probabilities of Tau pathology

(See Eklund et al. 2016)

Correction | na

Models & analysis

n/a	Involved in the study
☒	☐ Functional and/or effective connectivity
☒	☐ Graph analysis
☒	☐ Multivariate modeling or predictive analysis
